# Imaging of intracranial hemorrhage in photon counting computed tomography using virtual monoenergetic images

**DOI:** 10.1007/s00234-024-03308-z

**Published:** 2024-02-27

**Authors:** Denise Schoenbeck, Alexander Sacha, Julius Henning Niehoff, Christoph Moenninghoff, Jan Borggrefe, Sebastian Horstmeier, Alexey Surov, Iram Shahzadi, Ulrich Knappe, Jan Robert Kroeger, Arwed Elias Michael

**Affiliations:** 1 Department of Radiology, Neuroradiology and Nuclear Medicine, Johannes Wesling University Hospital, Ruhr University Bochum, Bochum, Germany; 2 Johannes Wesling University Hospital By Muehlenkreiskliniken AöR, Hans-Nolte-Straße 1, 32429 Minden, Germany; 3 Siemens Healthineers GmbH, Henkestr. 127, 91052 Erlangen, Germany; 4 Department of Neurosurgery, Johannes Wesling University Hospital, Ruhr University Bochum, Bochum, Germany

**Keywords:** Computed tomography, Photon counting computed tomography, Photon counting detector, Image quality, Intracranial hemorrhage

## Abstract

**Purpose:**

To determine the optimal virtual monoenergetic image (VMI) for detecting and assessing intracranial hemorrhage in unenhanced photon counting CT of the head based on the evaluation of quantitative and qualitative image quality parameters.

**Methods:**

Sixty-three patients with acute intracranial hemorrhage and unenhanced CT of the head were retrospectively included. In these patients, 35 intraparenchymal, 39 intraventricular, 30 subarachnoidal, and 43 subdural hemorrhages were selected. VMIs were reconstructed using all available monoenergetic reconstruction levels (40–190 keV). Multiple regions of interest measurements were used for evaluation of the overall image quality, and signal, noise, signal-to-noise-ratio (SNR), and contrast-to-noise-ratio (CNR) of intracranial hemorrhage. Based on the results of the quantitative analysis, specific VMIs were rated by five radiologists on a 5-point Likert scale.

**Results:**

Signal, noise, SNR, and CNR differed significantly between different VMIs (*p* < 0.001). Maximum CNR for intracranial hemorrhage was reached in VMI with keV levels > 120 keV (intraparenchymal 143 keV, intraventricular 164 keV, subarachnoidal 124 keV, and subdural hemorrhage 133 keV). In reading, no relevant superiority in the detection of hemorrhage could be demonstrated using VMIs above 66 keV.

**Conclusion:**

For the detection of hemorrhage in unenhanced CT of the head, the quantitative analysis of the present study on photon counting CT is generally consistent with the findings from dual-energy CT, suggesting keV levels just above 120 keV and higher depending on the location of the hemorrhage.

However, on the basis of the qualitative analyses, no reliable statement can yet be made as to whether an additional VMI with higher keV is truly beneficial in everyday clinical practice.

**Supplementary Information:**

The online version contains supplementary material available at 10.1007/s00234-024-03308-z.

## Introduction

Intracranial hemorrhage is a significant medical event that accounts for up to 15% of strokes and has a mortality rate of 40% within 1 month of occurrence [1]. For the diagnosis of non-traumatic as well as traumatic intracranial hemorrhage, unenhanced CT of the head is the method of choice offering rapid image acquisition and wide availability [2]. Diagnostic imaging is crucial for the physician in charge to determine the exact location and extent of the hemorrhage, as well as to assess the risk of impending brain damage and thus guide the patient’s treatment.

Previous studies have shown that dual-energy CT (DECT) improves image quality and lesion characterization through virtual monoenergetic imaging (VMI) [3, 4]. Furthermore, it is known from earlier studies using DECT that intracranial hemorrhages have a maximum contrast-to-noise ratio in VMI 120 keV [4]. Photon counting CT (PCCT) currently represents the latest technical development in clinical CT [5], and its underlying detector technology might offer advantages in the field of neuroradiological imaging. To date, it has not been studied at which VMI hemorrhage can be best delineated in Photon Counting CT.

The purpose of this study was to evaluate quantitative and qualitative image quality parameters of virtual monoenergetic images of unenhanced CT of the head in patients with intracranial hemorrhage and to determine the optimal monoenergetic level for hemorrhage detection and assessment.

## Material and methods

### Patient population

Institutional review board approval was obtained. Informed consent was waived due to the retrospective study design. All patients who had undergone unenhanced CT of the head at our institution between September and December 2022 were identified, and studies with acute (≤ 7 days) intracranial hemorrhage were selected. Each CT had been performed with a clinical protocol and with medical indication. Examinations with pronounced motion artifacts or artifacts due to foreign material were excluded. Included data were anonymized.

### CT protocol and image acquisition

All CT scans were performed using the clinically approved photon-counting CT (NAEOTOM Alpha, software version Syngo CT VA50, Siemens Healthineers, Erlangen, Germany) with a spiral CT protocol. Patients were examined in a supine position with moderate flexion in the cervical spine to perform axial acquisition in the orbitomeatal plane. Single collimation was 0.4 mm, total collimation 38.4 mm, and pitch factor was 0.55 with a rotation time of 0.5 s. Tube voltage was 120 kVp, and the tube current was modulated due to the manufacturer’s program of dose modulation (image quality level 300). The matrix size was 512 × 512; the field of view (FOV) was optimized for the individual head size. The reconstruction kernel QR36 and the highest level of iterative reconstruction (Quantum Iterative Reconstruction, QIR level 4 out of 4) were used for the spectral data sets. Datasets were analyzed in the manufacturer-specific spectral workstation (Syngo.Via, VB60_B version, Siemens Healthineers, Erlangen, Germany). The images were reconstructed in axial view with a slice thickness of 3 mm and a slice increment of 3 mm.

### Quantitative image analysis

**Fig. 1:**
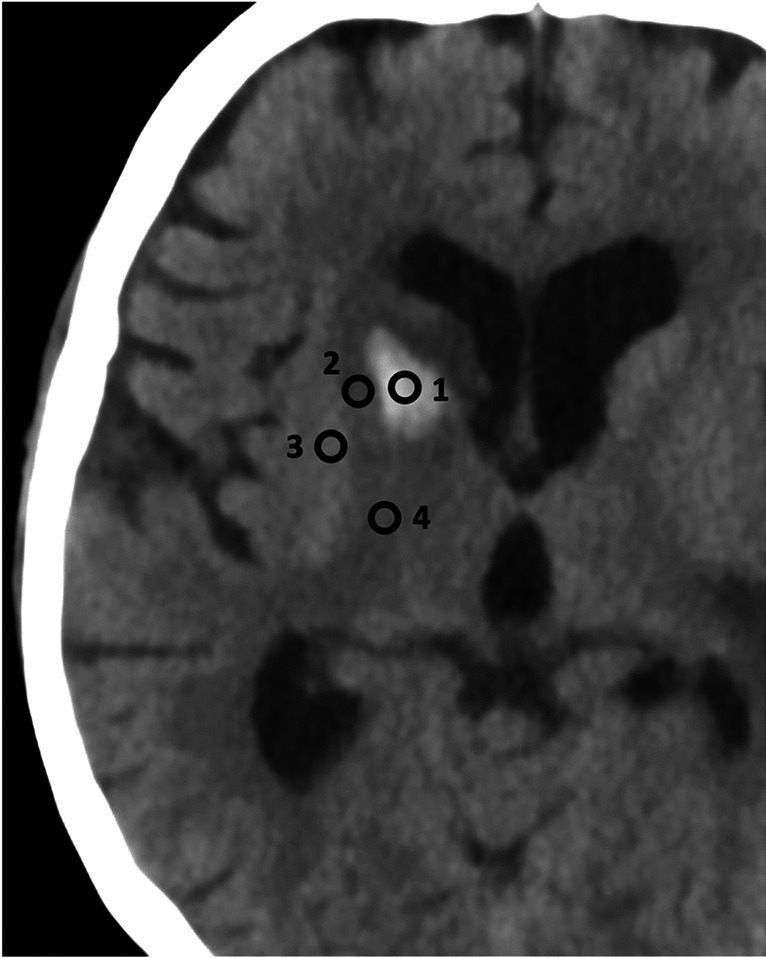
Position of ROIs in hemorrhage and adjacent tissue. ROIs were set in the acute hemorrhage (1), in the adjacent white matter with possible pathological changes such as edema (2), the nearest gray matter (3), and in the nearest nonpathologically altered white matter. All ROIs measured 4 mm.^2^. Image of a VMI 66 keV with windowing C40/W80

Sixteen different ROIs were used for the evaluation of the overall image quality in accordance with the latest PCCT studies [6]. Of those, nine ROIs involved the gray and white matter of the supratentorial cortex at different distances to the calvaria. The next six ROIs involved deep gray and deep white matter, namely the basal ganglia at two different locations superior and inferior, the thalamus and the immediately adjacent white matter in each case. Two ROIs were placed in the pons between the petrous bones to provide a measure of artifacts analogous to the preliminary studies [7]. The next ROIs were set in the acute hemorrhage, in the nearest gray matter, in the nearest nonpathologically altered white matter, and in the immediately adjacent white matter with possible pathological changes such as edema. Except for the ROI in the pons (200 mm^2^), all ROIs measured 4 mm^2^. Figure 1 shows the position of ROIs in hemorrhage and adjacent tissue.

For each ROI, the mean density and its standard deviation (SD) were measured for all available virtual monoenergetic images (151 different VMIs, from 40 to 190 keV in 1 keV steps). Parameters for assessing image quality were determined as described previously, in order to allow for comparison of study results [3, 7]. The signal of the ROIs was defined as the mean density in Hounsfield units (HU). Noise was defined as the SD of a ROI in HU. The signal-to-noise ratio (SNR) was calculated by dividing the mean density of the ROI (signal) by the corresponding SD of the ROI (noise). The contrast-to-noise ratio (CNR) was calculated as the quotient of the difference of the mean density of two adjacent ROIs with gray matter (GM) and white matter (WM) and the square root of the sum of the variance of both ROIs [7]. For calculating the CNR of the hemorrhage, the ROI in the hemorrhage and the ROI in the nearest gray matter were used.

### Qualitative image analysis

**Fig. 2:**
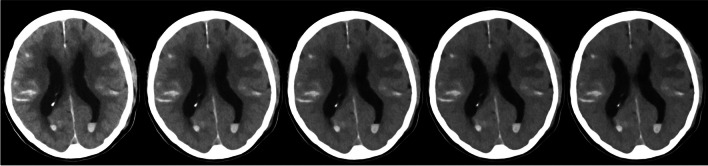
Selection of VMIs for the qualitative analysis. From left to right VMIs 66 keV, 80 keV, 100 keV, 124 keV, and 133 keV, all showing the same axial slice with subarachnoidal and intraventricular hemorrhage (windowing C40/W80)

For each hemorrhage type, 20 cases were selected for qualitative analysis in which the respective hemorrhage type occurred in isolation in selected slices. Based on the results of the quantitative analysis, five specific VMIs (66 keV, 80 keV, 100 keV, 124 keV, 133 keV) were rated by five radiologists on a standard workstation: one experienced neuroradiologist with 15 years of experience, two general radiologists with 9 and 7 years of experience, and two residents with 4 and 2 years of experience, respectively. Those readers evaluated the delineation of the hemorrhage using a 5-point Likert scale (from 1 = “difficulty in delineating the bleeding, uncertain diagnosis” to 5 = “excellent delineation, fully diagnostic”). All readers were blinded to the keV level; every VMI was presented with equal standard windowing (C40/W80). Figure 2 shows the selection of VMI for the qualitative analysis, for reasons of illustration with two hemorrhage types, namely subarachnoidal and intraventricular hemorrhage.

### Statistical analysis

Data curation, data processing, and statistical analyses were performed using the statistical software R (Version 4.1.0) and RStudio (Version 2023.03.0 + 386). The Shapiro–Wilk test was applied to test for normal distribution, the Levene test to check for homoscedasticity. When considering the individual ROIs, a one-way ANOVA or the Friedman test as the corresponding nonparametric method was used to investigate whether the image parameters of the individual keV levels differed. Resulting *p* values were corrected using the Bonferroni method. For post hoc testing with selected keV levels, e.g., the keV level with the maximum or minimum value of a parameter and the adjacent keV levels as well as a representative selection of the spectrum, the paired *t*-test or the nonparametric Wilcoxon signed-rank test was used. Again, the correction of the *p* values was done using the Bonferroni method.

For qualitative analysis, appropriate nonparametric procedures were used (Friedman test and Wilcoxon test). If not stated otherwise, all data are presented as mean ± standard deviation, including the ordinal Likert data due to the frequent use of those parameters in the literature.

## Results

### Patient population and radiation dose

In total, 63 patients (31 women, 32 men) with intracranial hemorrhage, mostly in several different localizations, were included in this study (1 scan had to be excluded due to motion artifacts and 7 scans due to artifacts caused by foreign material like coils, clips, CSF drainage). In this study population, 35 patients had intraparenchymal, 39 patients had intraventricular, 30 patients had subarachnoidal, and 43 patients had subdural hemorrhages. The mean age was 71.2 ± 14.1 years (range 27–96 years). The average CTDI_vol_ was 47.0 ± 4.24; mean dose length product was 753.0 ± 70.8 mGy*cm.

### General image quality

In almost all ROIs, the signal reached its maximum in the VMI 40 keV, only the ROIs in the deep white matter were an exception with the lowest signal at 40 keV. The noise was without exception, the highest at 40 keV and fell with increasing keV to its minimum between 120 and 150 keV depending on the ROI. A focal maximum of the CNR of gray and white matter differentiation was found at about 65 keV, this also was the absolute maximum in the ROIs below the calvaria up to a distance of 10 mm. In the other localizations, the CNR reached its maximum at keV > 110 keV. The cranial calvaria had a distinct influence on the signal of supratentorial cortical gray and white matter: the closer a ROI was to the calvaria, the higher the signal. A detailed description of the analysis of the general image quality can be found in the supplemental material.

### Signal, noise, and SNR of intracranial hemorrhage

The signal of intracranial hemorrhage reached its maximum in the VMI 40 keV in all four types of intracranial hemorrhages, and the differences between VMIs were statistically significant (corrected *p* < 0.001). The magnitude of the maximum signal was dependent on the distance of the hemorrhage from the cranial calvaria. Thus, subdural hemorrhages reached a signal up to 88.93 ± 14.06 HU, already the signal at 41 keV was significantly lower (corrected *p* < 0.001). In the other types of hemorrhages, the maximum signal was lower (see Table 1). In post hoc testing, a significant difference was not achieved for intraparenchymal hemorrhages after *p*-value correction (e.g., 40 keV compared to 42 keV uncorrected *p* = 0.048); for intraventricular hemorrhages and subarachnoid hemorrhages, there were significant differences compared to the signal at 41 keV (corrected *p* < 0.05). The lowest noise was found in all hemorrhage types in a VMI with a higher keV level. As with the ROIs used to determine overall image quality (see “General image quality” section), a further focal decrease in noise was seen between 60 and 70 keV. The minimum noise was reached at 115 keV for intraparenchymal hemorrhage and 142 keV for intraventricular blood. In hemorrhage types closer to the cranial calvaria—subarachnoid and subdural blood—the lowest noise was found tending to be at a higher keV (see Table 1).

**Table 1:** Signal, noise, and SNR of intracranial hemorrhage

Hemorrhage type	Max. signal (keV level)	Min. noise (keV level)	Max. SNR (keV level)
Intraparenchymal	67.86 ± 14.24 HU (40 keV)	3.06 ± 2.17 HU (115 keV)	39.35 ± 39.75 (136 keV)
Intraventricular	62.97 ± 13.55 HU (40 keV)	2.54 ± 2.22 HU (132 keV)	42.96 ± 42.43 (142 keV)
Subarachnoidal	74.15 ± 11.80 HU (40 keV)	2.31 ± 1.36 HU (142 keV)	40.62 ± 51.32 (153 keV)
Subdural	88.93 ± 14.06 HU (40 keV)	2.32 ± 1.39 HU (158 keV)	37.37 ± 30.06 (142 keV)

*SNR* signal-to-noise ratio, *HU* Hounsfield units, *keV* kiloelectron volt

**Fig. 3:**
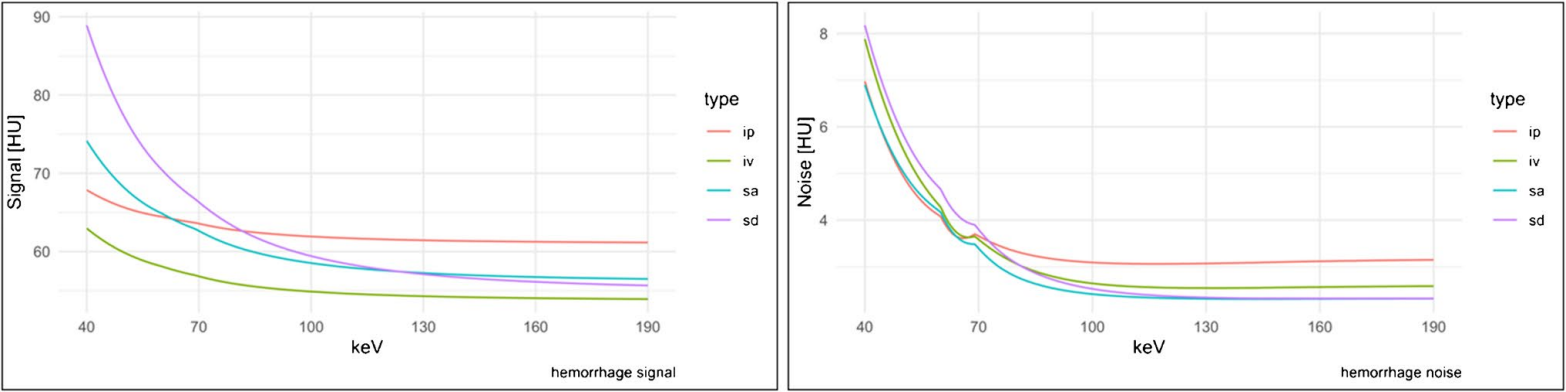
Signal and noise of intracranial hemorrhage. HU, Hounsfield units; keV, kiloelectron volt; ip, intraparenchymal hemorrhage; iv, intraventricular hemorrhage; sa, subarachnoidal hemorrhage; sd, subdural hemorrhage

The focal decrease in noise between 60 and 70 keV resulted in a focal increase in SNR. However, the maximum values of the SNR are found at higher keV, exemplified by 37.37 ± 30.06 at 142 keV (see Table 1) for subdural hemorrhage. The values for signal and noise are also displayed in Fig. 3.

### CNR of intracranial hemorrhage

The CNR in VMI with different keV levels differed significantly (*p* < 0.001). Due to the focal decrease in noise between 60 and 70 keV, there was also a small focal increase in CNR in this range. For intraparenchymal hemorrhage, the absolute maximum of CNR was 14.13 ± 15.61 in the VMI 143 keV; in post hoc testing, there was no significant difference for VMI with higher keV, and for VMI with a keV ≤ 105 keV, the CNR was significantly lower.

**Fig. 4:**
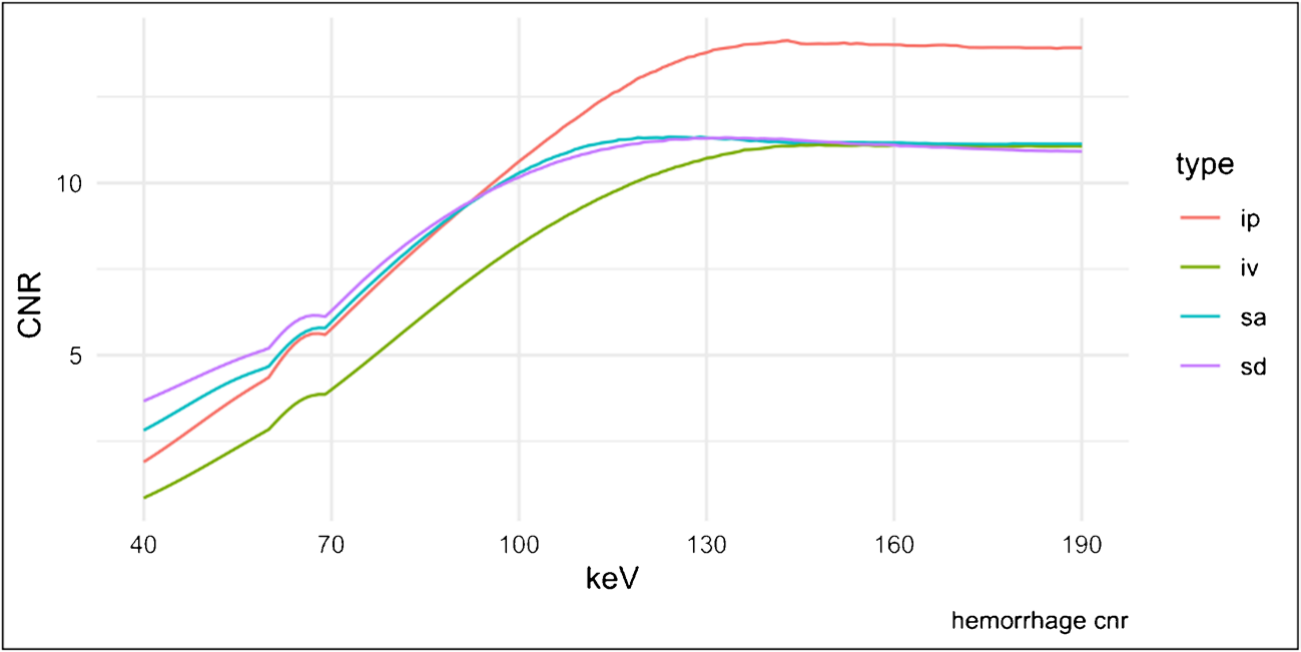
Contrast-to-noise-ratio of intracranial hemorrhage. CNR, contrast-to-noise-ratio; ip, intraparenchymal hemorrhage; iv, intraventricular hemorrhage; sa, subarachnoidal hemorrhage; sd, subdural hemorrhage. The CNR was calculated as the quotient of the difference of the mean density of the ROI in the hemorrhage and the ROI in the nearest gray matter divided by the square root of the sum of the variance of both ROIs

For intraventricular blood, the maximum CNR was 11.10 ± 10.61 in VMI 164 keV. Again, there was no significant difference from the VMI with higher keV, but the CNR is significantly lower from 105 keV and lower keV levels. For subarachnoid hemorrhage, the maximum CNR was 11.33 ± 9.98 (124 keV), and for subdural hemorrhage, 11.31 ± 10.39 (133 keV). For both types of hemorrhage, there was no significant difference for VMI with higher keV; for subarachnoid hemorrhage, the CNR is significantly lower for VMI ≤ 105 keV and for subdural ≤ 95 keV. See Fig. 4 for detailed information on CNR.

### Qualitative image analysis

Based on the results of the quantitative analysis, five VMIs were used for the qualitative image analysis. First, VMI 66 keV with the maximum gray-white differentiation known from previous studies [6], which is therefore currently also used for clinical purpose. Second, VMIs 124 and 133 keV with the maximum CNR for subarachnoid and subdural hemorrhages, which may be more of a diagnostic challenge than intraparenchymal or intraventricular hemorrhages due to their proximity to the calvaria. Finally, VMI 80 keV and VMI 100 keV were added as intermediate steps.

**Fig. 5:**
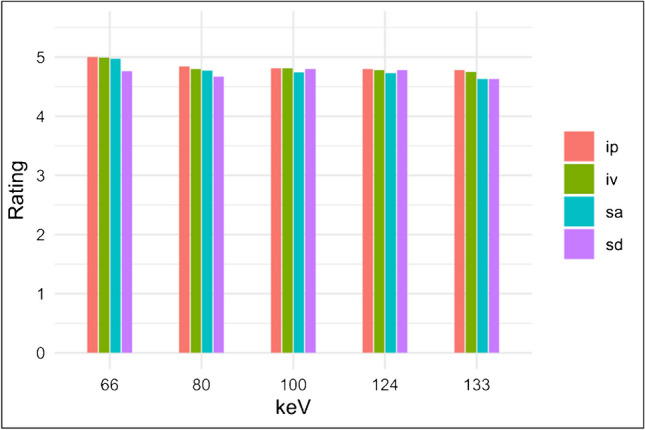
Results of qualitative image analysis. Rating: 5-point Likert scale from 1 = “difficulty in delineating the bleeding, uncertain diagnosis” to 5 = “excellent delineation, fully diagnostic.” The graphic shows the average rating despite the ordinal scale. keV, kiloelectron volt; ip, intraparenchymal hemorrhage; iv, intraventricular hemorrhage; sa, subarachnoidal hemorrhage; sd, subdural hemorrhage

For all hemorrhage types, this resulted in a high rating with a median = 5 in each reconstruction and in each hemorrhage type. There were small and also significant differences (Friedman test *p* < 0.001) between the individual ratings, but these were not considered relevant (lowest rating of 4.63 ± 0.49 for subarachnoid and subdural hemorrhages at 133 keV), so post hoc testing was not performed. See Fig. 5 for further information on the results of the qualitative image analysis.

## Discussion

This study investigated, in which VMI reconstructed from non-enhanced CT examinations of the head intracranial hemorrhage are depicted best. Regarding the general image quality, the results were consistent with those of recent studies [6]. In the quantitative analysis, the VMIs at 143 keV for intraparenchymal, at 164 keV for intraventricular, at 124 keV for subarachnoid, and at 133 keV for subdural hemorrhage offered the highest CNR. In the qualitative analysis, however, the readers were able to delineate hemorrhage on selected VMIs with high keV (124 and 133 keV) about as well as on the VMI with low keV (66 keV), which is commonly used clinically because of maximum gray-white differentiation [6]. The delineation capability here was generally reported to be excellent.

To the best of our knowledge, this is the first study addressing the detection of intracranial hemorrhage in PCCT scans of the head. However, the results of the quantitative analysis are generally consistent with the results of previous studies in dual-energy CT. In dual-energy CT, VMIs reconstructed from non-enhanced CT of the head at 65 and 120 keV offers optimized contrast between gray and white matter and reduction of beam hardening artifacts caused by the skull, respectively [3, 8]. Neuhaus et al. determined that image noise as well as subcalvarian artifacts decreased significantly on VMI at 120 keV. Regarding the differentiation between gray and white matter, Neuhaus et al. observed the highest CNR values on VMI at 40 keV for Dual Layer CT [8]. Similar findings were recently obtained for photon counting CT: optimal contrast between gray and white matter was found at 66 keV in quantitative and 60 keV in qualitative analysis, respectively. At the same time, image noise was significantly reduced at higher keV levels [6].

With regard to optimal keV levels for the detection of intracranial hemorrhage, data are scarce and only available for dual-energy CT. Bondanapally et al. inferred that virtual high-monochromatic (190 keV) images in combination with 120 keV images can provide optimal results for the detection of intracranial hemorrhage in patients suspected of traumatic brain injury [9]. Lennartz et al. suggest that the level 120 keV should be selected if discrete subarachnoid, subdural, and epidural hemorrhages are to be detected because beam hardening artifacts and noise in the subcalvar space are lowest here; low keV levels (40 keV) may allow more precise localization of small amounts of hemorrhage within the basal ganglia [4]. Cho et al. investigated in a small cohort to what extent spectral analysis and different VMIs can be helpful in distinguishing contrast agent uptake from bleeding. Here, low keV VMIs were found to be particularly helpful in detecting iodine uptake. However, the question, of which keV levels are useful for the detection of intracranial hemorrhage in unenhanced CT of the head was not addressed in this study [10]. For the detection of hemorrhage, the quantitative analysis of the present study on photon counting CT is generally consistent with these findings from dual-energy CT in unenhanced CT of the head, yielding toward keV levels just above 120 keV and higher depending on the type of hemorrhage.

The fact that the results of the qualitative analysis in this study do not fully agree with the findings of the quantitative analysis and the findings from the dual-energy CT studies may be of particular interest. However, a detailed analysis shows that the differences are not as distinct as they may appear. In their qualitative analysis, Lennartz et al. investigated low keV such as 40 and 55 keV [4]. At keV levels higher than 55 keV, the same median rating for the detection of hemorrhages was found; above 100 keV, a significant difference to conventional polyenergetic reconstructions occurred. The VMIs 40 and 55 keV were rated the lowest regarding the delineation of hemorrhage. In our study, VMIs with keV < 66 keV was not included in the subjective evaluation, as the quantitative results showed the superiority of VMIs with higher keV, and it is known from preliminary studies that VMIs < 60 keV are currently also inferior in terms of general image quality [6]. So far, a detailed comparison of the various VMIs with each other has not been performed. Thus, concretely, the only conclusion drawn from these data in the literature is that for hemorrhage detection, VMIs with a keV level > 55 keV are superior to VMIs with lower keV levels. This finding does not contradict our study, in which the lowest keV VMI was reconstructed with 66 keV. Also, in the study of Bodanapally et al., the superiority of the VMI 190 keV compared to VMI 120 keV was only confirmed for certain subtypes of hemorrhages; for subarachnoid hemorrhage, VMI 120 keV performed better, and other VMIs were not included in the comparative reading [9]. Therefore, even in the literature on conventional dual-energy CT, it has not been demonstrated conclusively that a certain VMI is superior for hemorrhage detection in clinical practice when the standard VMI is prepared at 66 keV or similar.

However, in view of the clear results of the quantitative analysis, the question arises as to why the superiority of the VMIs with higher keV could not be shown in the qualitative analysis. First, while evaluating the VMIs, the readers did not have a time limit to detect the hemorrhage and were then asked to evaluate the delineation. Second, scans without intracranial hemorrhage were not presented, so there was always certainty about the presence of hemorrhage. It is conceivable that a VMI at higher keV—particularly in a stressful situation and with time pressure—might pay off in the detection of intracranial hemorrhage. Thus, further studies are needed to clarify this matter.

This study has certain limitations. The retrospective study design generally carries the risk of selection or information bias. MRI was not available for all study participants and was therefore not chosen as a reference standard. Regarding the qualitative assessment, there was a lack of true blinding because the different VMI could be easily identified by the readers on the basis of noise perception or subjective image impression—this, however, seems inevitable. In addition, the readers knew that bleeding was present in every CT scan. Only VMIs with the fourth level of iterative reconstruction were used, although an influence of the iterative reconstruction on the results cannot be excluded. Finally, the analysis did not differentiate between supra- and infratentorial hemorrhage, so the proximity to bony structures was only indirectly taken into account by the type of bleeding.

In summary, our study provides a robust quantitative analysis regarding the imaging of intracranial hemorrhage in unenhanced CT of the head in PCCT with results that seem comprehensible based on the findings in DECT. However, based on the qualitative analysis, no reliable statement can yet be made as to whether a VMI with higher keV is truly beneficial in everyday clinical practice. Based on the data presented here, the standard VMI with 66 keV or similar is not significantly inferior to the VMI with higher keV. Further studies are needed to find the optimal VMI for detecting intracranial hemorrhage.

### Supplementary Information

Below is the link to the electronic supplementary material.

Supplementary file1 (DOCX 2024 KB)

## Data Availability

Data are available on reasonable request.

## References

[1] van Asch CJ, Luitse MJ, Rinkel GJ, van der Tweel I, Algra A, Klijn CJ (2010). Incidence, case fatality, and functional outcome of intracerebral haemorrhage over time, according to age, sex, and ethnic origin: a systematic review and meta-analysis. Lancet Neurol.

[2] Greenberg SM, Ziai WC, Cordonnier C, Dowlatshahi D, Francis B, Goldstein JN, Hemphill JC, Johnson R, Keigher KM, Mack WJ (2022). Guideline for the management of patients with spontaneous intracerebral hemorrhage: a guideline from the American Heart Association/American Stroke Association. Stroke.

[3] Pomerantz SR, Kamalian S, Zhang D, Gupta R, Rapalino O, Sahani DV, Lev MH (2013). Virtual monochromatic reconstruction of dual-energy unenhanced head CT at 65–75 keV maximizes image quality compared with conventional polychromatic CT. Radiology.

[4] Lennartz S, Laukamp KR, Neuhaus V, Große Hokamp N, Le Blanc M, Maus V, Kabbasch C, Mpotsaris A, Maintz D, Borggrefe J (2018). Dual-layer detector CT of the head: initial experience in visualization of intracranial hemorrhage and hypodense brain lesions using virtual monoenergetic images. Eur J Radiol.

[5] Flohr T, Schmidt B (2023). Technical basics and clinical benefits of photon-counting CT. Invest Radiol.

[6] Michael AE, Schoenbeck D, Woeltjen MM, Boriesosdick J, Kroeger JR, Moenninghoff C, Horstmeier S, Niehoff JH, Kabbasch C, Goertz L (2023). Nonenhanced photon counting CT of the head : impact of the keV level, iterative reconstruction and calvaria on image quality in monoenergetic images. Clin Neuroradiol.

[7] Michael AE, Boriesosdick J, Schoenbeck D, Woeltjen MM, Saeed S, Kroeger JR, Horstmeier S, Lennartz S, Borggrefe J, Niehoff JH (2022). Image-quality assessment of polyenergetic and virtual monoenergetic reconstructions of unenhanced CT scans of the head: initial experiences with the first photon-counting CT approved for clinical use. Diagnostics.

[8] Neuhaus V, Abdullayev N, Große Hokamp N, Pahn G, Kabbasch C, Mpotsaris A, Maintz D, Borggrefe J (2017). Improvement of image quality in unenhanced dual-layer CT of the head using virtual monoenergetic images compared with polyenergetic single-energy CT. Invest Radiol.

[9] Bodanapally UK, Archer-Arroyo KL, Dreizin D, Shanmuganathan K, Schwartzbauer G, Li G, Fleiter TR (2019). Dual-energy computed tomography imaging of head: virtual high-energy monochromatic (190 keV) images are more reliable than standard 120 kV images for detecting traumatic intracranial hemorrhages. J Neurotrauma.

[10] Cho SB, Baek HJ, Ryu KH, Moon JI, Choi BH, Park SE, Bae K, Jeon KN, Kim DW (2017). Initial clinical experience with dual-layer detector spectral CT in patients with acute intracerebral haemorrhage: a single-centre pilot study. PLoS ONE.

