# Supplementary material for: Imaging of intracranial hemorrhage in photon counting computed tomography using virtual monoenergetic images
Source: Neuroradiology. 2024 Feb 27;66(5):729–36. doi: 10.1007/s00234-024-03308-z (PMC11031477; doi:10.1007/s00234-024-03308-z)
Supplement: Supplementary file 1 — Supplementary file1 (DOCX 2024 KB) [file 234_2024_3308_MOESM1_ESM.docx]

This document provides Supplemental Material for the paper titled:

## Imaging of Intracranial Hemorrhage in Photon Counting Computed Tomography Using Virtual Monoenergetic Images

The statistical analysis was performed using the statistical software R (R Core Team, 2021), graphics were created using the R package ggplot2 (Wickham, 2009).

______________________

Core Team. (2021). R: A Language and Environment for Statistical Computing.

Available at: https://www.R-project.org/

Wickham, H. (2009). ggplot2: Elegant Graphics for Data Analysis: Springer New York.

# Abbreviations

| PCCT | Photon Counting Computed Tomography |
| --- | --- |
| CCT | Cranial Computed Tomography |
| DECT | Dual Energy Computed Tomography |
| VMI | Virtual Monoenergetic Image |
| QIR | Quantum Iterative Reconstruction |

| gray_subc | ROI (1): cortical gray matter below the calvaria |
| --- | --- |
| gray_5mm | ROI (2): cortical gray matter 5 mm below the calvaria |
| white_5mm | ROI (3): cortical white matter 5 mm below the calvaria |
| gray_10mm | ROI (4): cortical gray matter 10 mm below the calvaria |
| white_10mm | ROI (5): cortical white matter 10 mm below the calvaria |
| gray_15mm | ROI (6): cortical gray matter 15 mm below the calvaria |
| white_15mm | ROI (7): cortical white matter 15 mm below the calvaria |
| gray_20mm | ROI (8): cortical gray matter 20 mm below the calvaria |
| white_20mm | ROI (9): cortical white matter 20 mm below the calvaria |
| gray_caputhigh | ROI (10): gray matter in the superior caudate head |
| white_anthigh | ROI (11): white matter adjacent to ROI (6) in the superior internal capsule |
| gray_caputlow | ROI (12): gray matter in the inferior caudate head |
| white_antlow | ROI (13): white matter adjacent to ROI (8) in the anterior callosum |
| gray_thal | ROI (14): gray matter in the posterior thalamus |
| white_post | ROI (15): white matter in the posterior internal capsule |
| pons | ROI (16): white matter in the pons between the petrous bones |
| Q4 | Level 4 of QIR, maximal iterative reconstruction |
|  |  |
| ip | intraparenchymal (hemorrhage) |
| iv | intraventricular (hemorrhage) |
| sa | subarachnoidal (hemorrhage) |
| sd | subdural (hemorrhage) |

# Analysis of Image Quality

In the coming sections, the results of the analyses of the individual parameters of the image quality are shown. First, the signal is presented, then the noise; after a short presentation of the Signal to Noise Ratio (SNR), the results regarding the Contrast to Noise Ratio (CNR) are finally presented.

All image quality parameters are shown for each ROI as a function of the keV level.

For each ROI signal and noise are displayed in a single graph, the values are given in Hounsfield Units (HU). The "/" sign on the y-axis does not mean "divided by", but is intended to indicate that both the values of signal and noise are shown on a single axis.

**ROI (1): Gray Matter below the Calvaria**


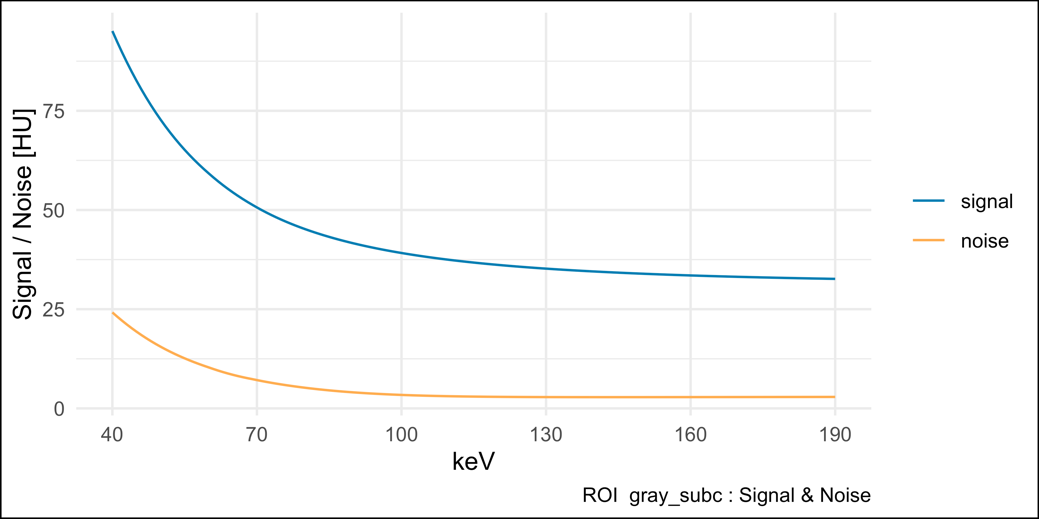


There are significant differences between signal and noise at different keV levels (Friedman test corrected p < 0.001). Maximal signal is 95.11 ± 24.23 HU in the reconstruction with 40 keV. Minimal noise is 2.83 ± 2.00 HU in the reconstruction with 142 keV.


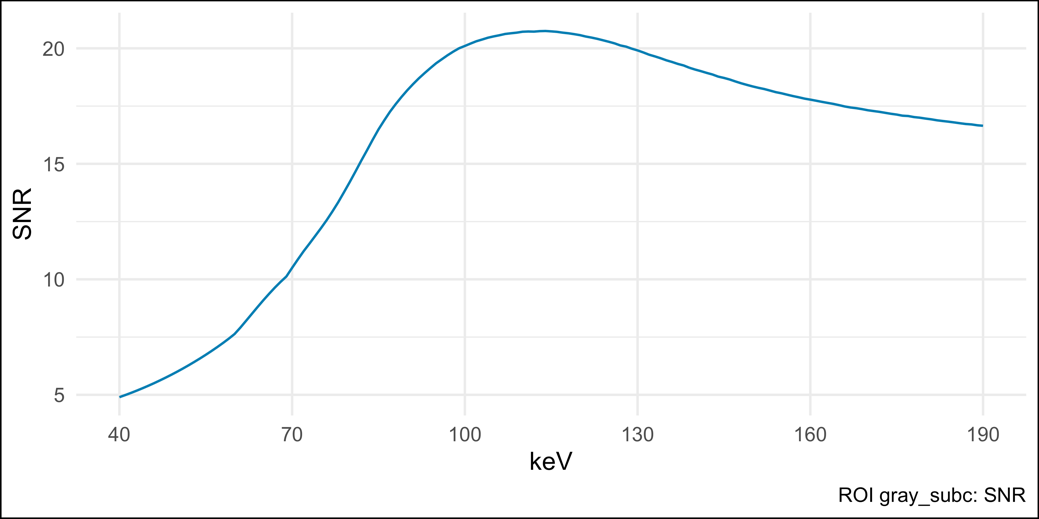


There are significant differences between the SNR at different keV levels (Friedman test corrected p < 0.001). The maximal SNR is 20.75 ± 14.74 in the reconstruction with 114 keV.


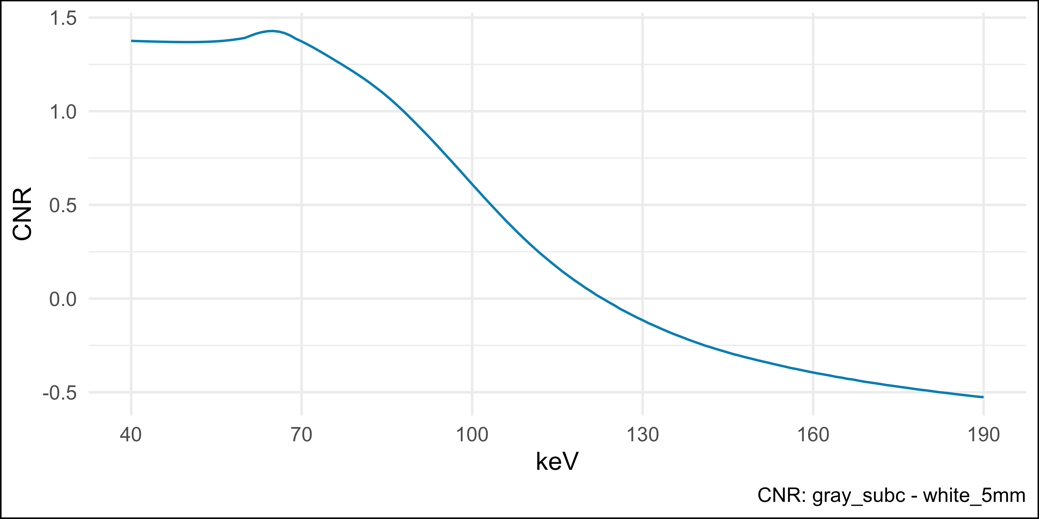


For the calculation of the CNR, the ROI (1) in the gray matter immediately below the calvaria was paired with the ROI (3) in the white matter at a distance of 5 mm from the calvaria. There are significant differences between the CNR at different keV levels (Friedman test corrected p < 0.001). The maximal CNR is 95.11 ± 24.23 HU in the reconstruction with 40 keV. The values of signal, noise, SNR and CNR of all keV levels as well as the results of the post hoc testing can be viewed in an a supplemental table (data_image_quality.xlsx).

**ROI (2) and ROI (3): Gray and White Matter 5 mm below the Calvaria**


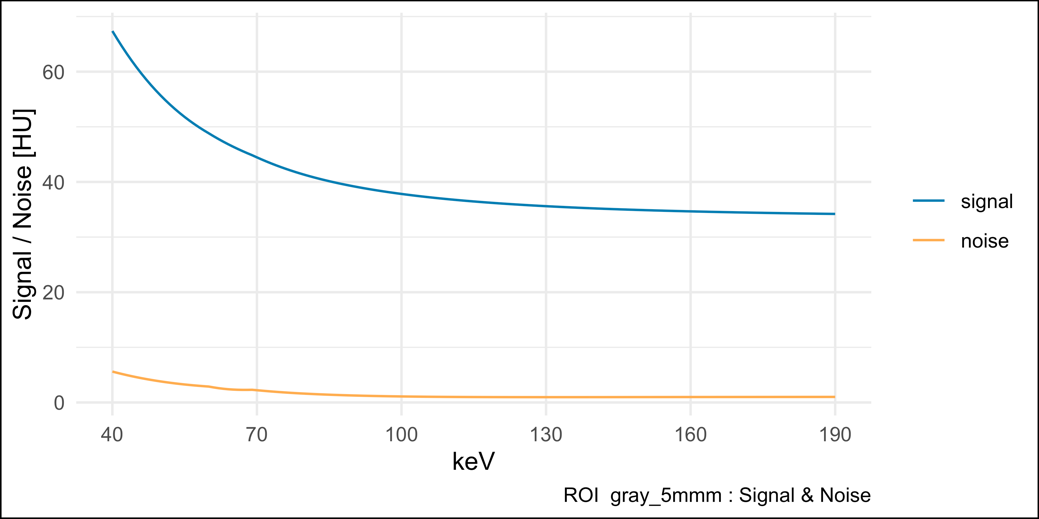


There are significant differences between signal and noise at different keV levels (Friedman test corrected p < 0.001). The maximal signal is 67.38 ± 12.56 HU in the reconstruction with 40 keV. The minimal noise is 0.96 ± 0.78 HU in the reconstruction with 130 keV.


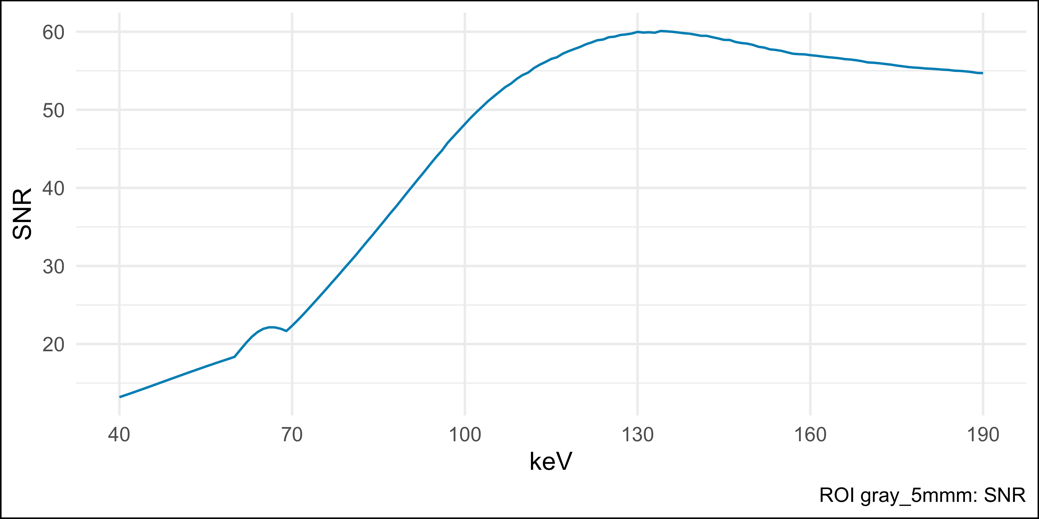


There are significant differences between the SNR at different keV levels (Friedman test corrected p < 0.001). The maximal SNR is 60.09 ± 41.12 in the reconstruction with 134 keV.


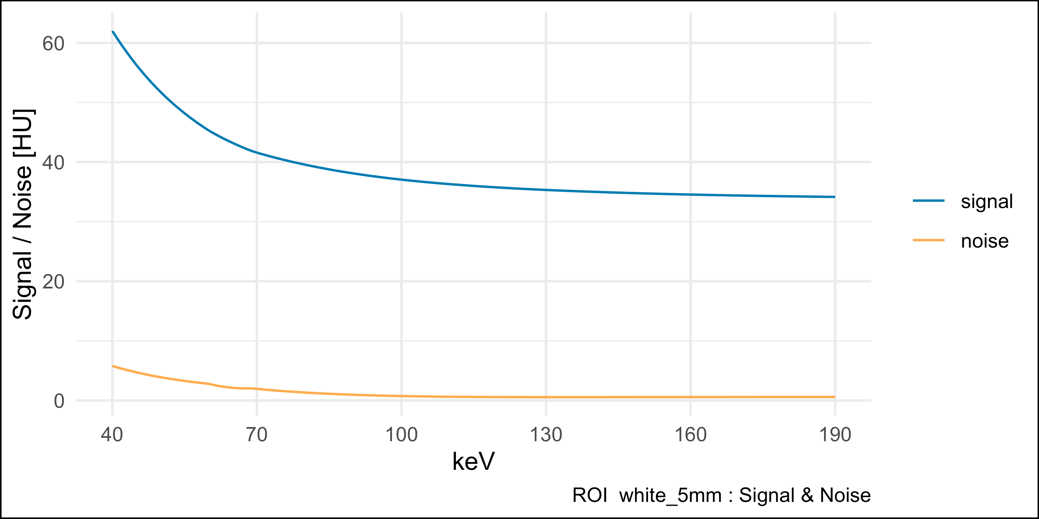


There are significant differences between signal and noise at different keV levels (Friedman test corrected p < 0.001). The maximal signal is 61.99 ± 11.76 HU in the reconstruction with 40 keV. The minimal noise is 0.55 ± 0.29 HU in the reconstruction with 135 keV.


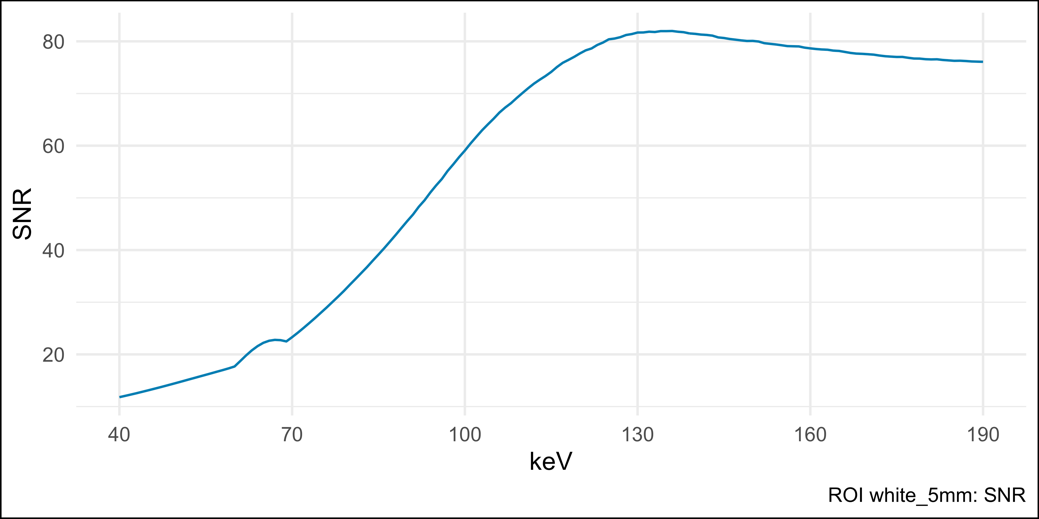


There are significant differences between the SNR at different keV levels (Friedman test corrected p < 0.001). The maximal SNR is 81.98 ± 40.26 HU in the reconstruction with 136 keV.


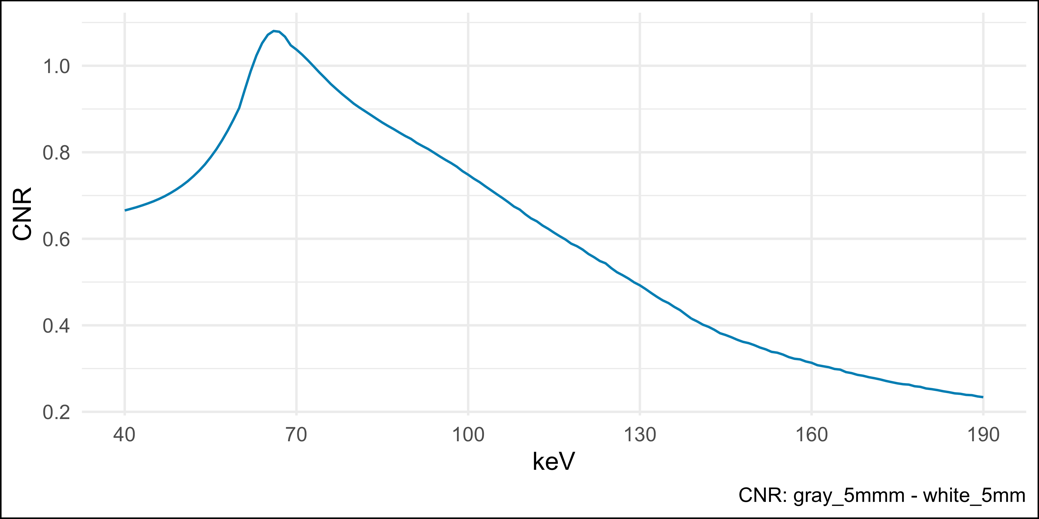


There are significant differences between the CNR at different keV levels (Friedman test corrected p < 0.001). The maximal CNR is 1.43 ± 0.82 in the reconstruction with 65 keV.

The values of signal, noise, SNR and CNR of all keV levels as well as the results of the post hoc testing can be viewed in an a supplemental table (data_image_quality.xlsx).

**ROI (4) and ROI (5): Gray and White Matter 10 mm below the Calvaria**


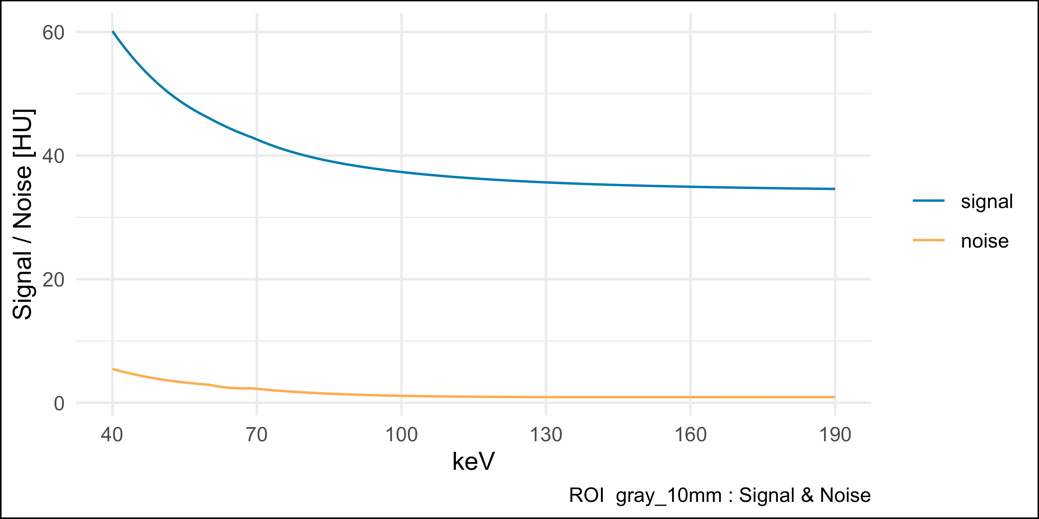


There are significant differences between signal and noise at different keV levels (Friedman test corrected p < 0.001). The maximal signal is 60.15 ± 8.22 HU in the reconstruction with 40 keV. The minimal noise is 0.91 ± 0.72 HU in the reconstruction with 141 keV.


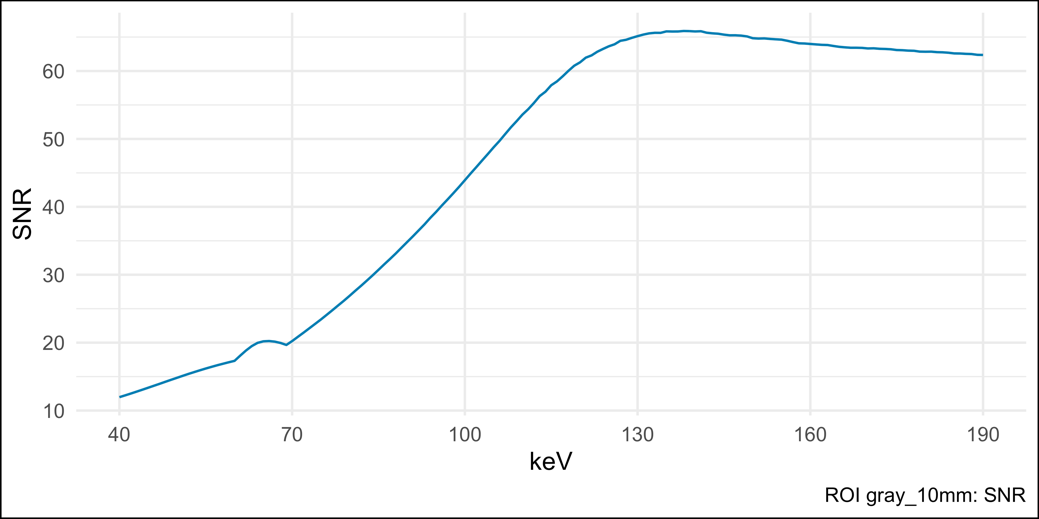


There are significant differences between the SNR at different keV levels (Friedman test corrected p < 0.001). The maximal SNR is 65.89 ± 42.82 in the reconstruction with 138 keV.


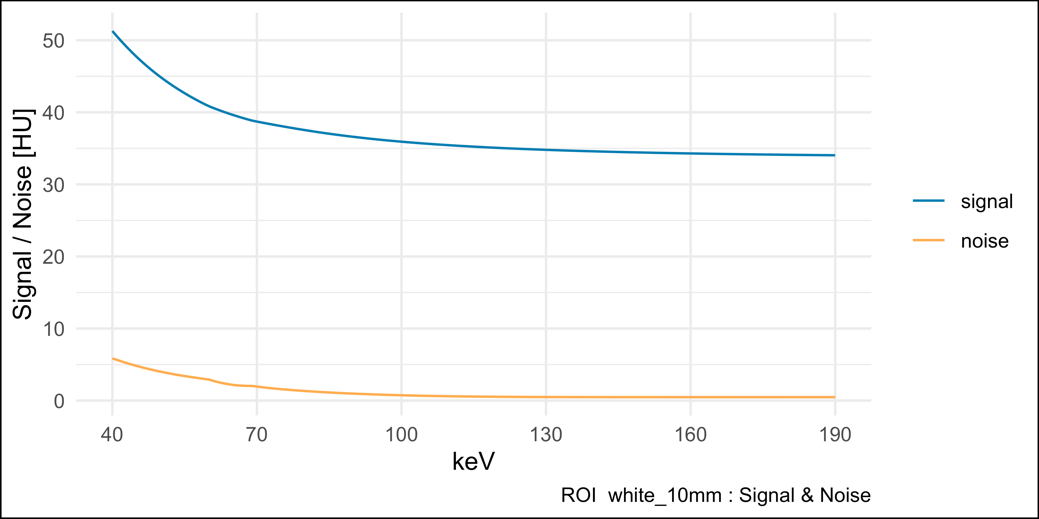


There are significant differences between signal and noise at different keV levels (Friedman test corrected p < 0.001). The maximal signal is 51.29 ± 8.31 HU in the reconstruction with 40 keV. The minimal noise is 0.48 ± 0.20 HU in the reconstruction with 166 keV.


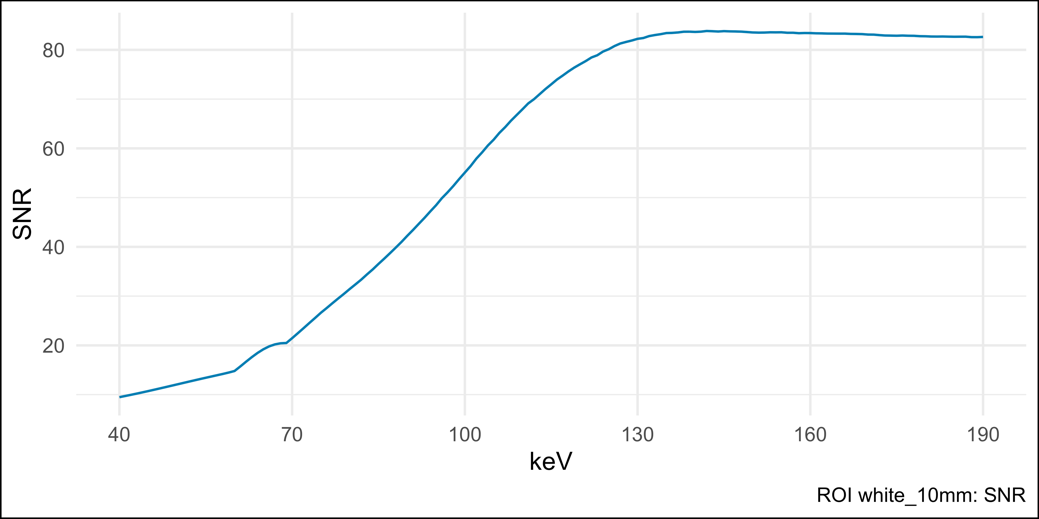


There are significant differences between the SNR at different keV levels (Friedman test corrected p < 0.001). The maximal SNR is 83.83 ± 30.51 in the reconstruction with 142 keV.


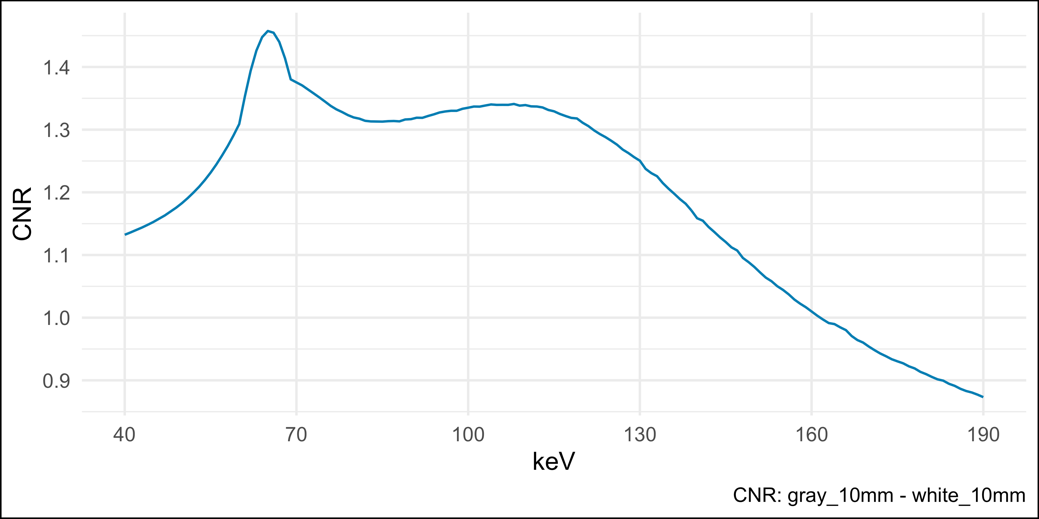


There are significant differences between the CNR at different keV levels (Friedman test corrected p < 0.001). The maximal CNR is 1.46 ± 1.09 in the reconstruction with 65 keV.

The values of signal, noise, SNR and CNR of all keV levels as well as the results of the post hoc testing can be viewed in an a supplemental table (data_image_quality.xlsx).

**ROI (6) and ROI (7): Gray and White Matter 15 mm below the Calvaria**


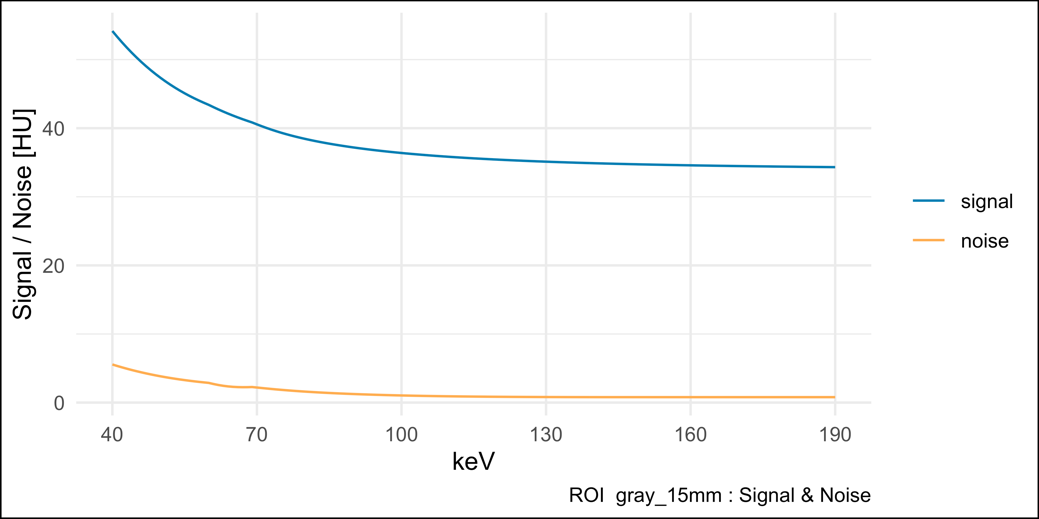


There are significant differences between signal and noise at different keV levels (Friedman test corrected p < 0.001). The maximal signal is 54.17 ± 8.18 HU in the reconstruction with 40 keV. The minimal noise is 0.79 ± 0.68 HU in the reconstruction with 157 keV.


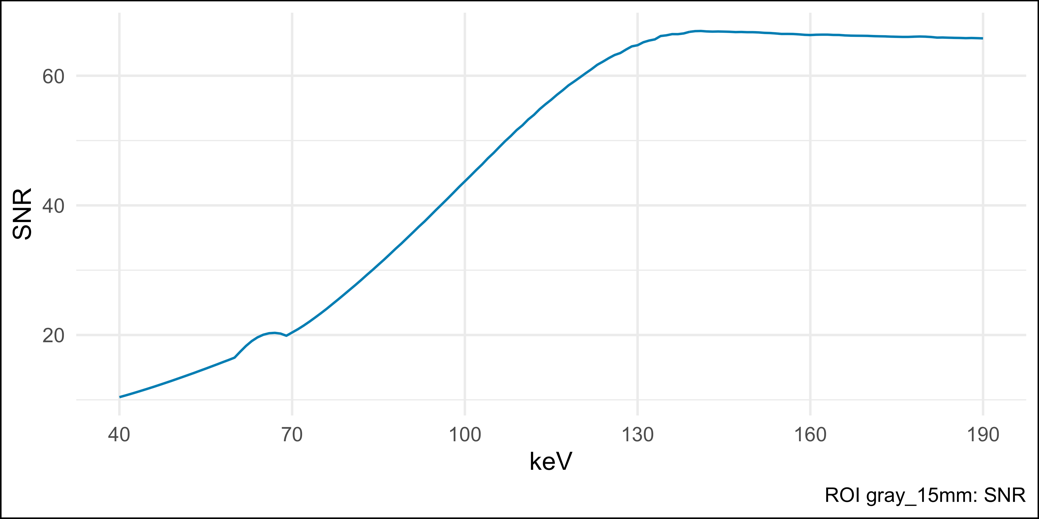


There are significant differences between the SNR at different keV levels (Friedman test corrected p < 0.001). The maximal SNR is 66.91 ± 37.91 in the reconstruction with 141 keV.


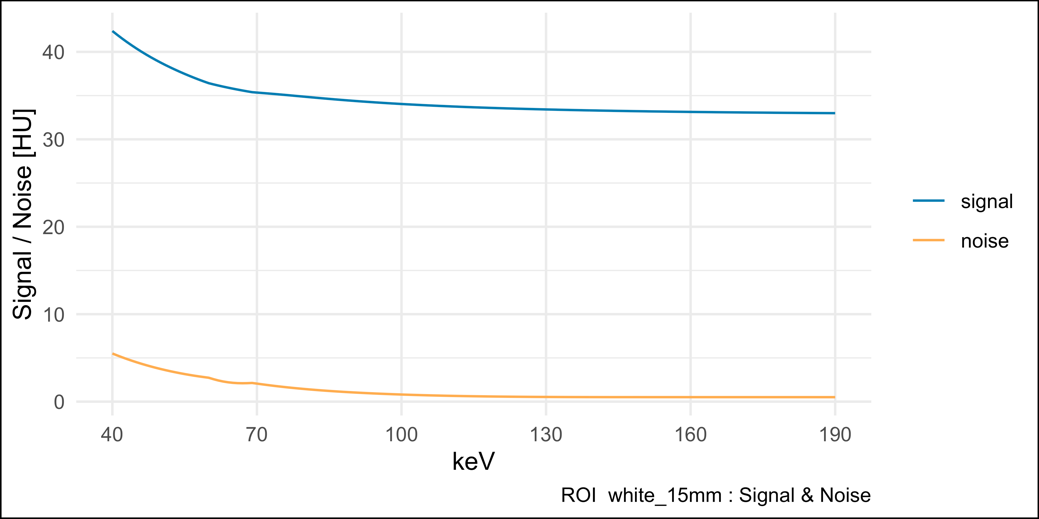


There are significant differences between signal and noise at different keV levels (Friedman test corrected p < 0.001). The maximal signal is 42.39 ± 7.28 HU in the reconstruction with 40 keV. The minimal noise is 0.51 ± 0.26 HU in the reconstruction with 187 keV.


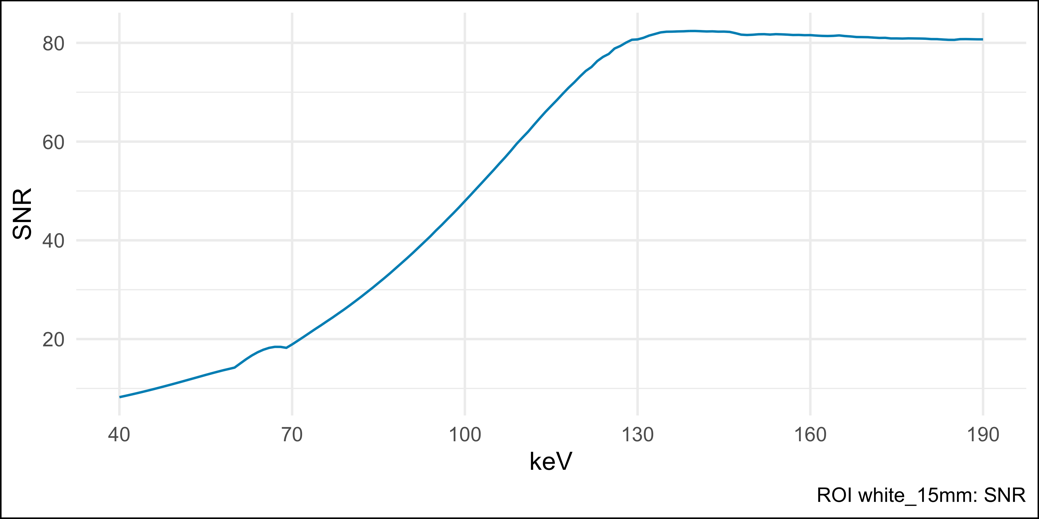


There are significant differences between the SNR at different keV levels (Friedman test corrected p < 0.001). The maximal SNR is 82.41 ± 41.00 in the reconstruction with 140 keV.


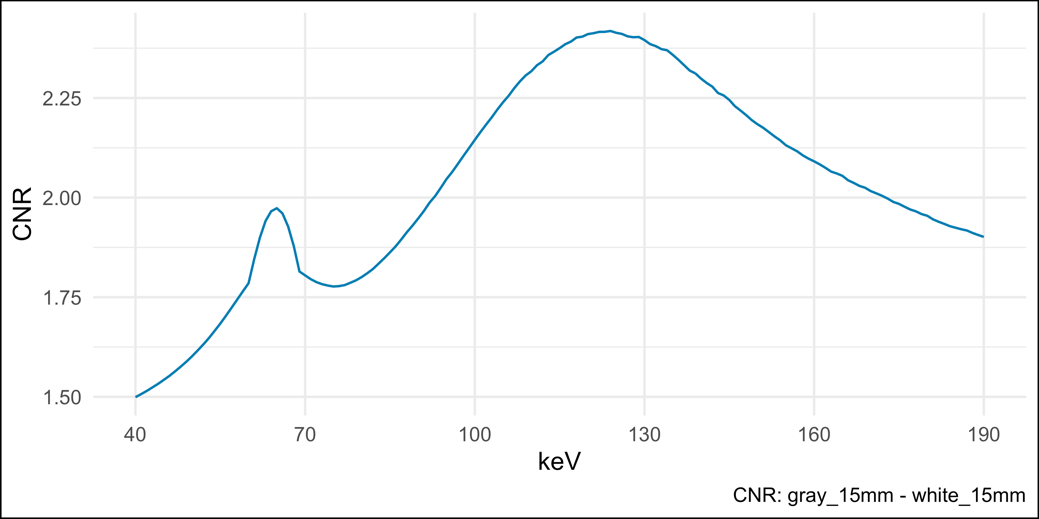


There are significant differences between the CNR at different keV levels (Friedman test corrected p < 0.001). The maximal CNR is 2.42 ± 2.75 in the reconstruction with 124 keV.

The values of signal, noise, SNR and CNR of all keV levels as well as the results of the post hoc testing can be viewed in an a supplemental table (data_image_quality.xlsx).

**ROI (8) and ROI (9): Gray and White Matter 20 mm below the Calvaria**


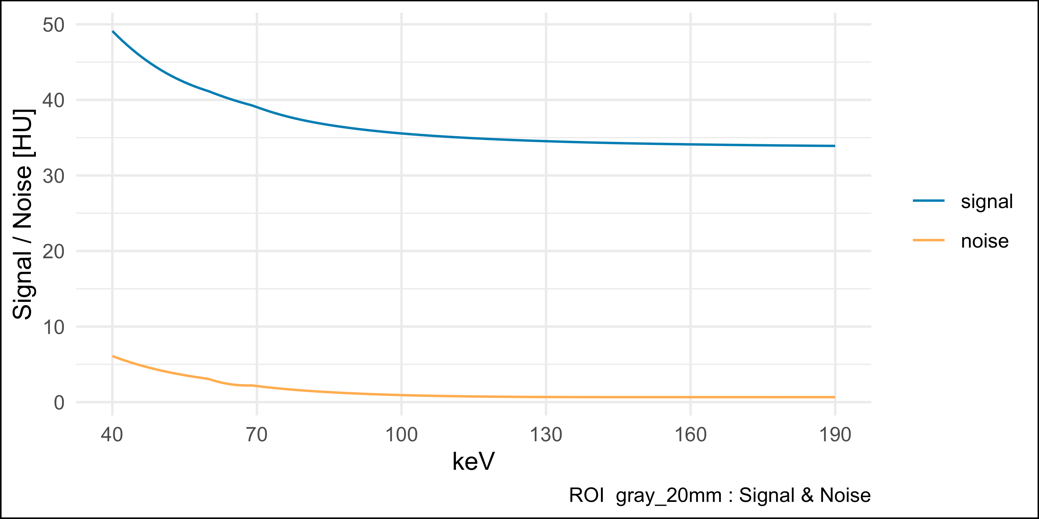


There are significant differences between signal and noise at different keV levels (Friedman test corrected p < 0.001). The maximal signal is 49.11 ± 7.24 HU in the reconstruction with 40 keV. The minimal noise is 0.66 ± 0.6 HU in the reconstruction with 190 keV.


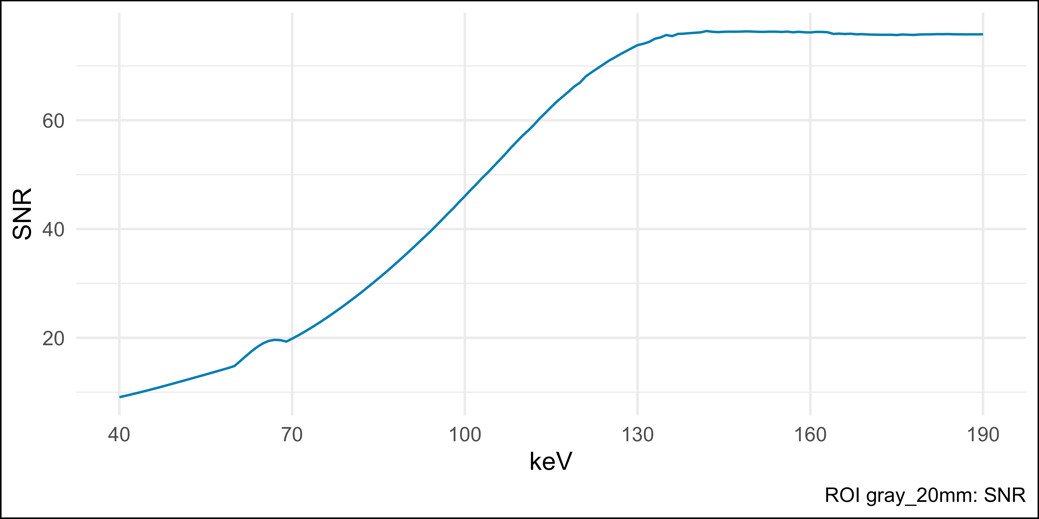


There are significant differences between the SNR at different keV levels (Friedman test corrected p < 0.001). The maximal SNR is 76.42 ± 41.3 in the reconstruction with 142 keV.


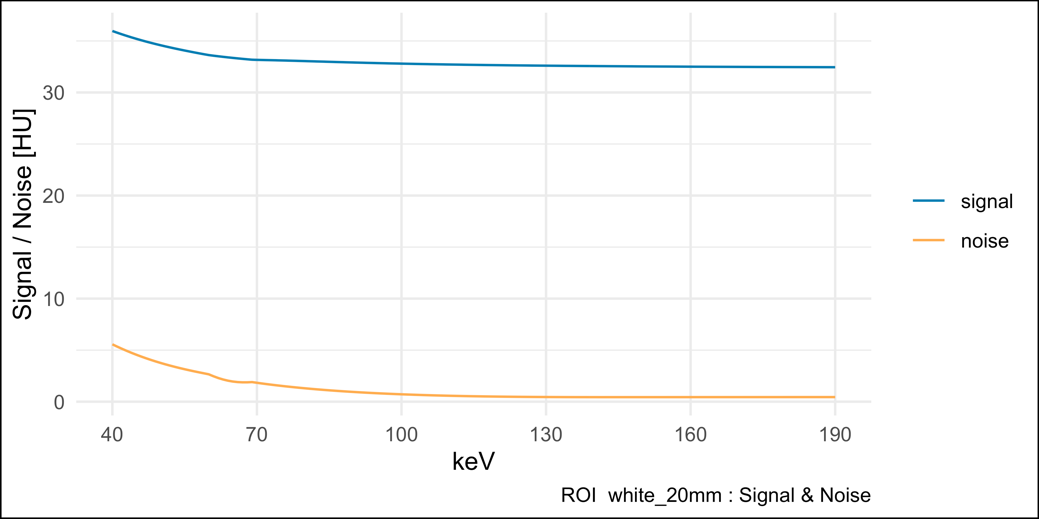


There are significant differences between signal and noise at different keV levels (Friedman test corrected p < 0.001). The maximal signal is 35.97 ± 6.08 HU in the reconstruction with 40 keV. The minimal noise is 0.44 + 0.25 HU in the reconstruction with 141 keV.


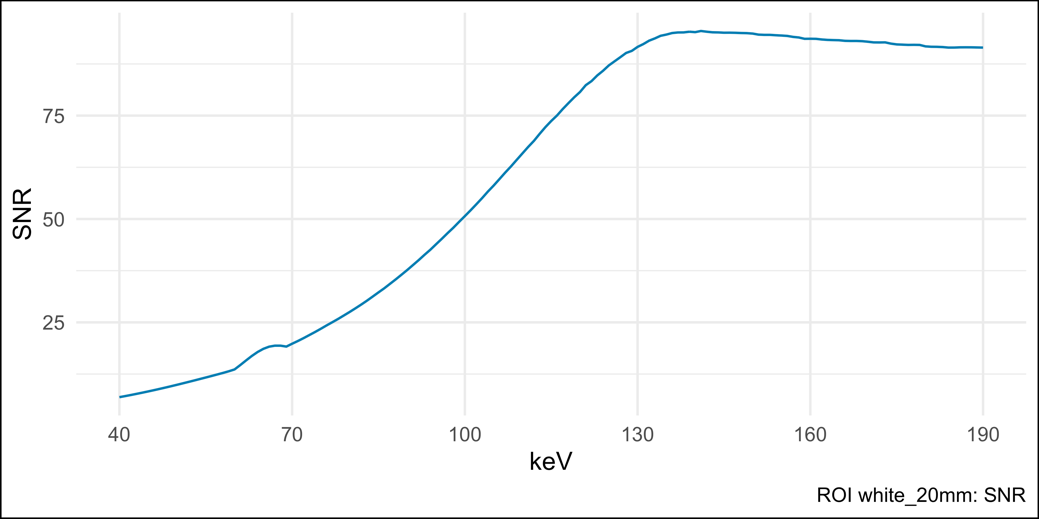


There are significant differences between the SNR at different keV levels (Friedman test corrected p < 0.001). The maximal SNR is 95.47 ± 47.42 in the reconstruction with 141 keV.


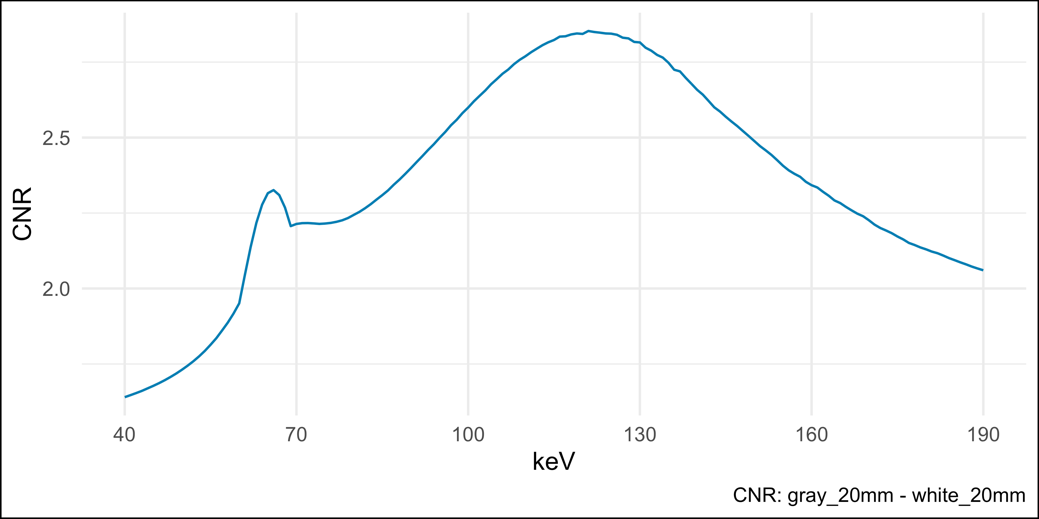


There are significant differences between the CNR at different keV levels (Friedman test corrected p < 0.001). The maximal CNR is 2.85 ± 2.36 in the reconstruction with 121 keV.

The values of signal, noise, SNR and CNR of all keV levels as well as the results of the post hoc testing can be viewed in an a supplemental table (data_image_quality.xlsx).

**ROI (10) and ROI (11): Gray and White Matter at the Superior Caudate Head**


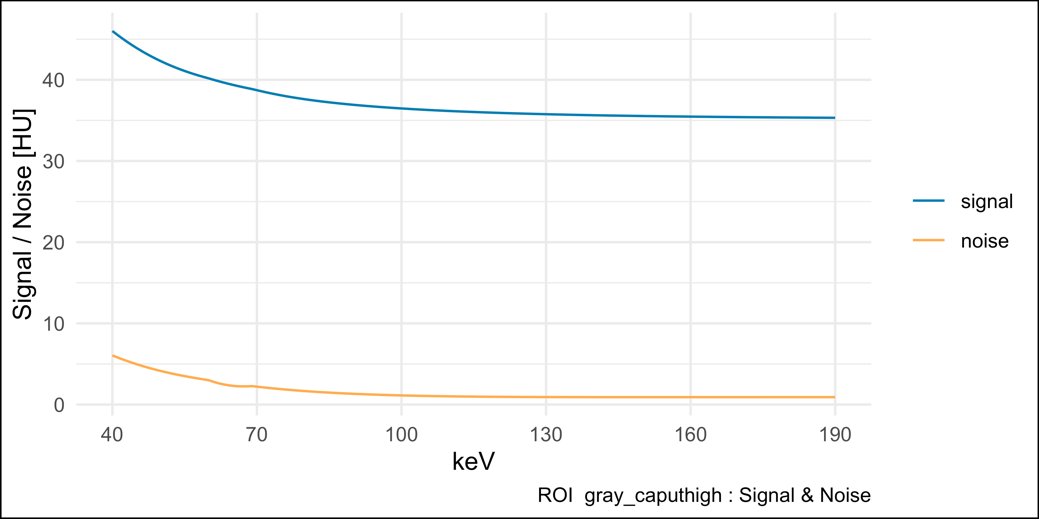


There are significant differences between signal and noise at different keV levels (Friedman test corrected p < 0.001). The maximal signal is 46.02 ± 5.77 HU in the reconstruction with 40 keV. The minimal noise is 0.91 ± 0.79 HU in the reconstruction with 144 keV.


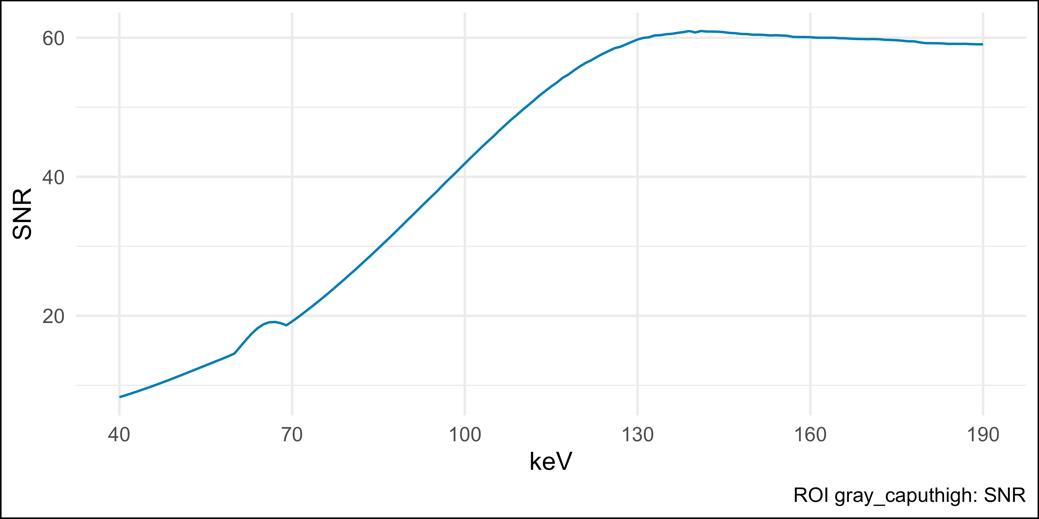


There are significant differences between the SNR at different keV levels (Friedman test corrected p < 0.001). The maximal SNR is 60.97 ± 38.16 in the reconstruction with 139 keV.


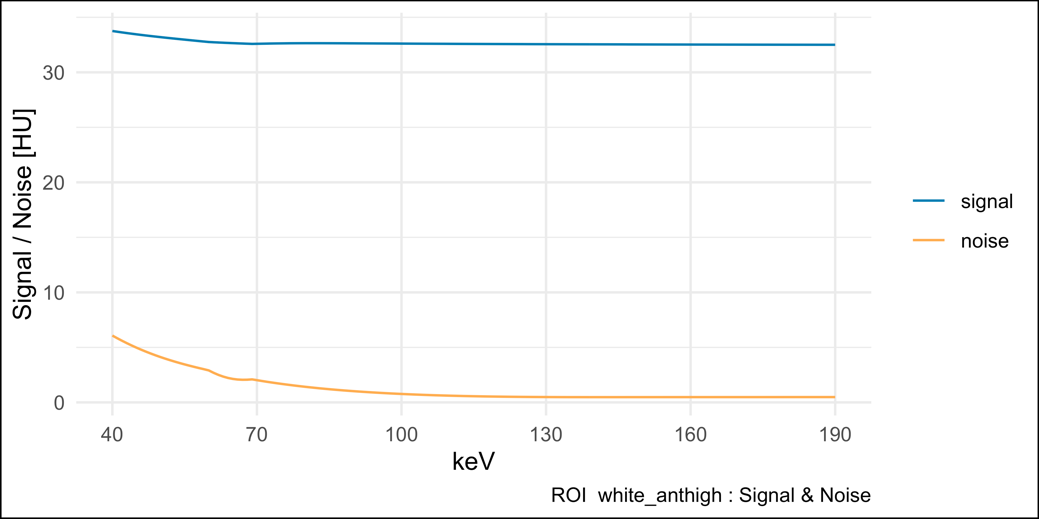


There are significant differences between signal and noise at different keV levels (Friedman test corrected p < 0.001). The maximal signal is 33.75 ± 6.61 HU in the reconstruction with 40 keV. The minimal noise is 0.48 ± 0.32 HU in the reconstruction with 139 keV.


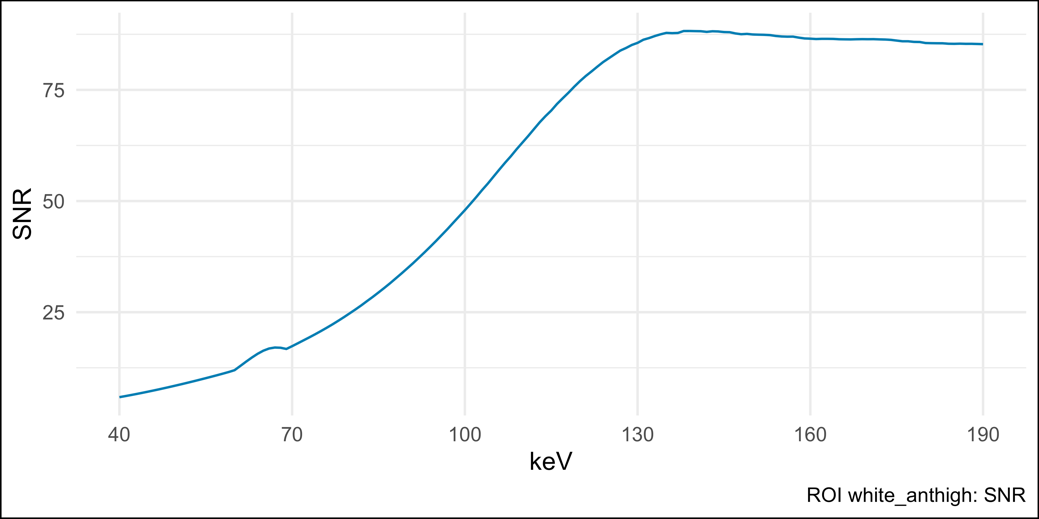


There are significant differences between the SNR at different keV levels (Friedman test corrected p < 0.001). The maximal SNR is 88.24 ± 40.06 in the reconstruction with 139 keV.


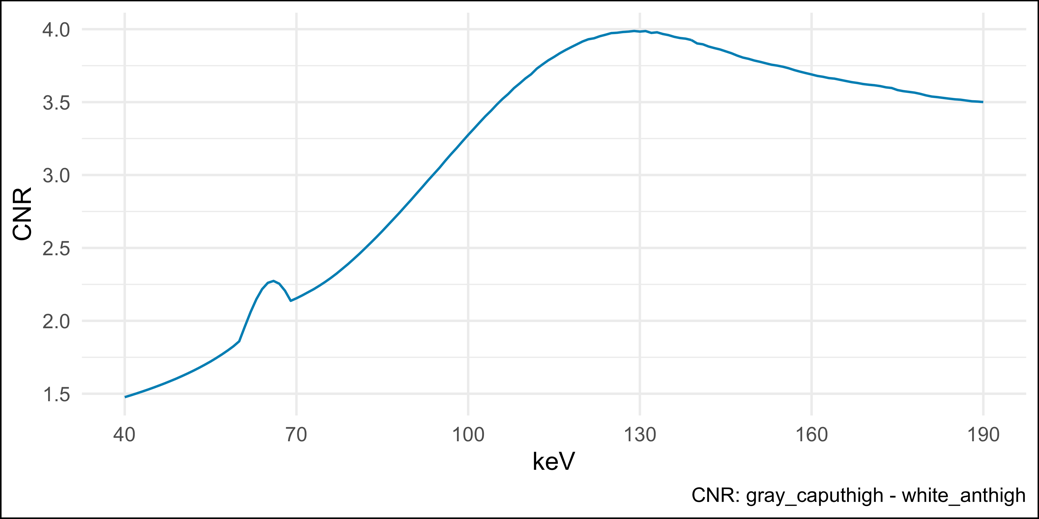


There are significant differences between the CNR at different keV levels (Friedman test corrected p < 0.001). The maximal CNR is 3.99 ± 3.29 in the reconstruction with 129 keV.

The values of signal, noise, SNR and CNR of all keV levels as well as the results of the post hoc testing can be viewed in an a supplemental table (data_image_quality.xlsx).

**ROI (12) and ROI (13): Gray and White Matter at the Inferior Caudate Head**


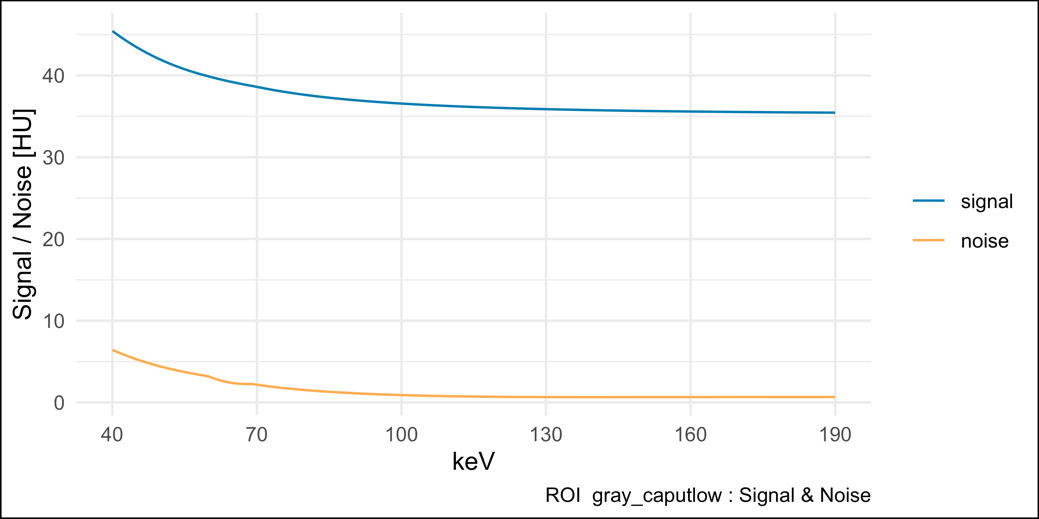


There are significant differences between signal and noise at different keV levels (Friedman test corrected p < 0.001). The maximal signal is 45.43 ± 6.63 HU in the reconstruction with 40 keV. The minimal noise is 0.65 ± 0.63 HU in the reconstruction with 141 keV.


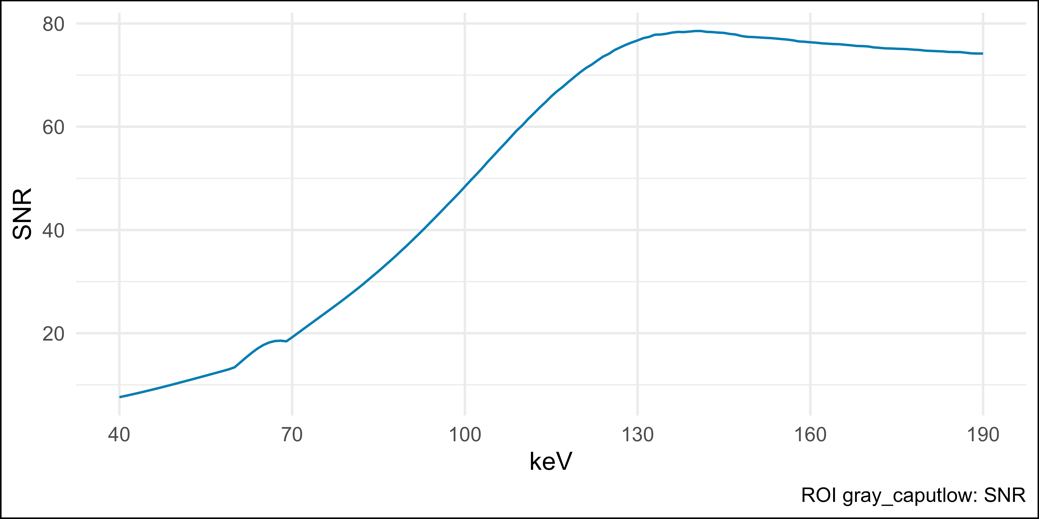


There are significant differences between the SNR at different keV levels (Friedman test corrected p < 0.001). The maximal SNR is 78.55 ± 37.14 in the reconstruction with 141 keV.


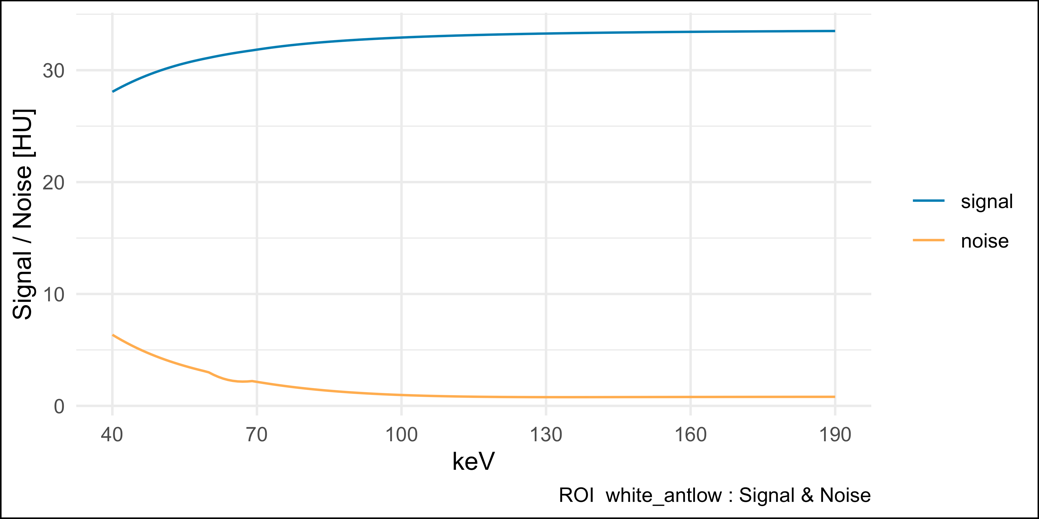


There are significant differences between signal and noise at different keV levels (Friedman test corrected p < 0.001). The maximal signal is 33.5 ± 1.97 HU in the reconstruction with 190 keV. The minimal noise is 0.78 ± 0.64 HU in the reconstruction with 133 keV.


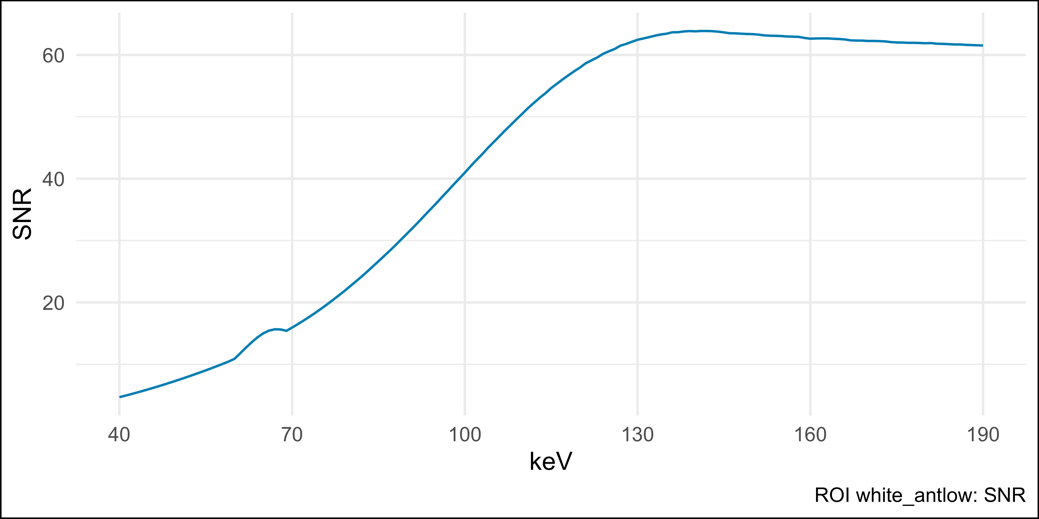


There are significant differences between the SNR at different keV levels (Friedman test corrected p < 0.001). The maximal SNR is 63.87 ± 34.71 in the reconstruction with 142 keV.


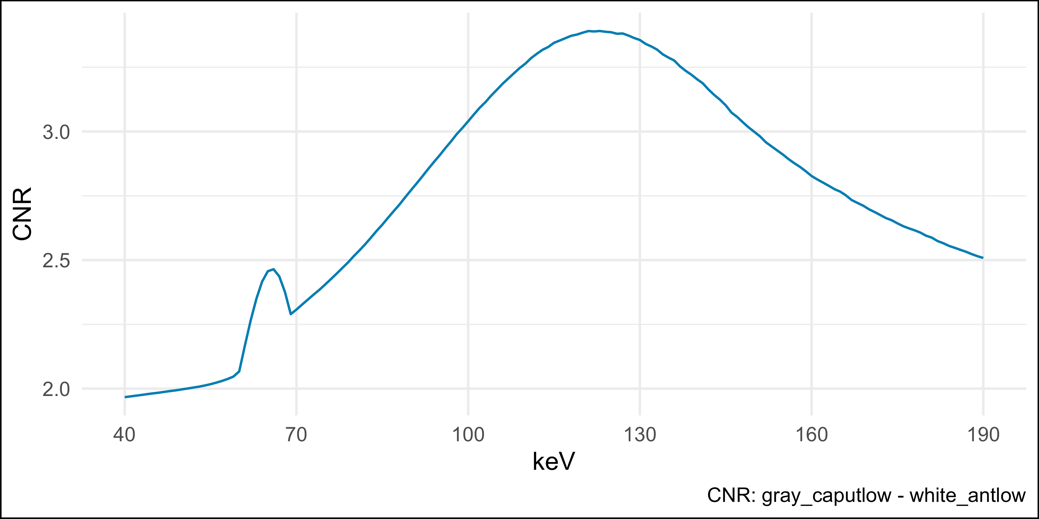


There are significant differences between the CNR at different keV levels (Friedman test corrected p < 0.001). The maximal CNR is 3.39 ± 2.84 in the reconstruction with 123 keV.

The values of signal, noise, SNR and CNR of all keV levels as well as the results of the post hoc testing can be viewed in an a supplemental table (data_image_quality.xlsx).

**ROI (14) and ROI (15): Gray and White Matter at Thalamus**


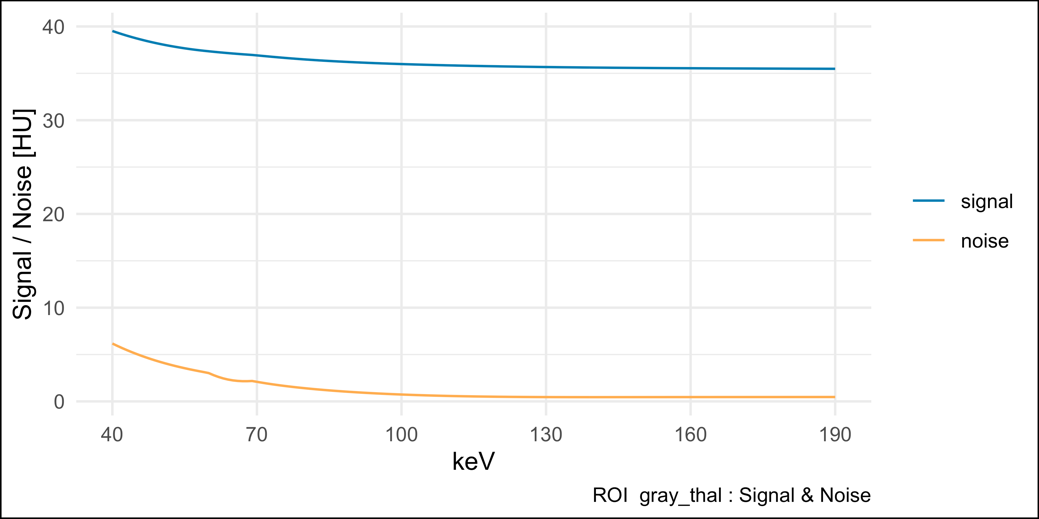


There are significant differences between signal and noise at different keV levels (Friedman test corrected p < 0.001). The maximal signal is 39.52 ± 6.1 HU in the reconstruction with 40 keV. The minimal noise is 0.45 ± 0.16 HU in the reconstruction with 140 keV.


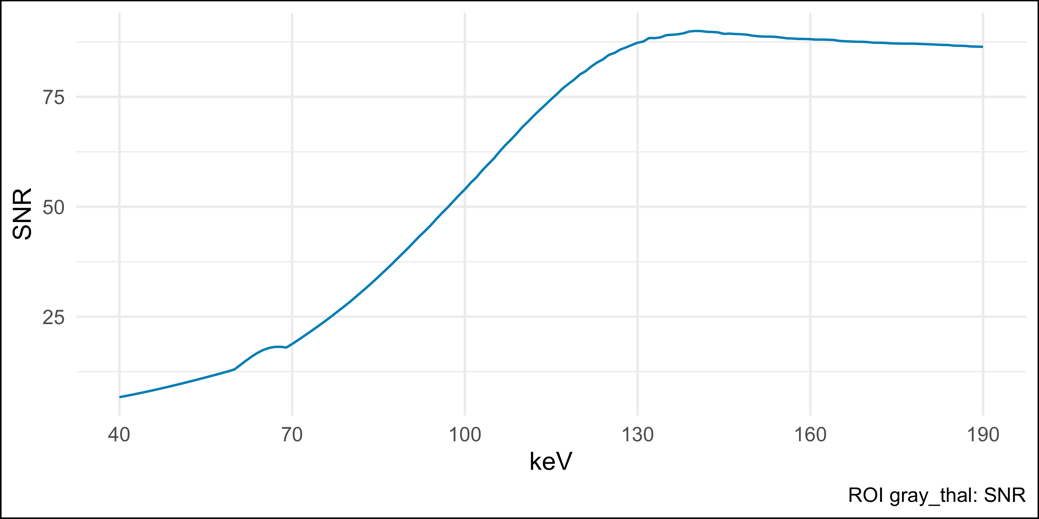


There are significant differences between the SNR at different keV levels (Friedman test corrected p < 0.001). The maximal SNR is 89.98 ± 35.26 in the reconstruction with 140 keV.


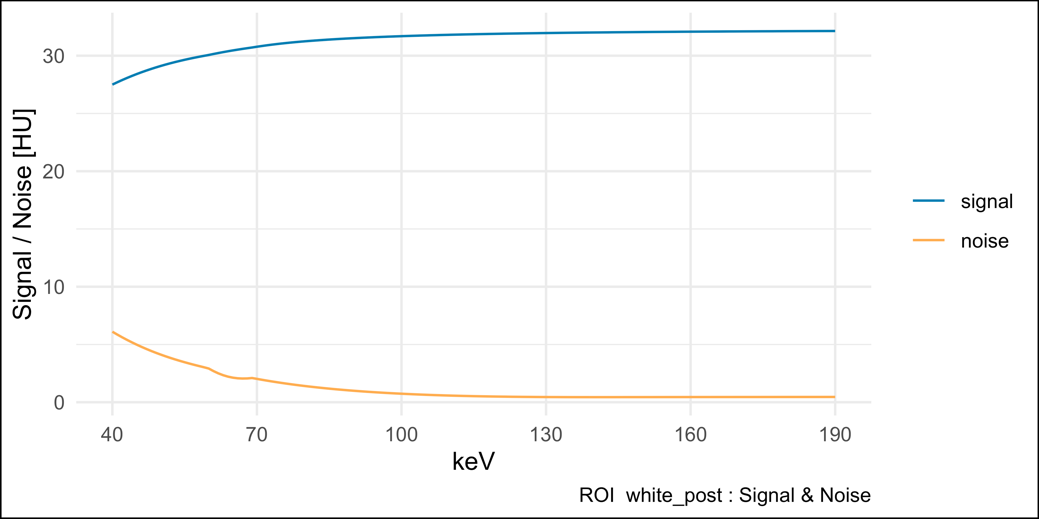


There are significant differences between signal and noise at different keV levels (Friedman test corrected p < 0.001). The maximal signal is 27.5 ± 6.24 HU in the reconstruction with 40 keV. The minimal noise is 0.44 ± 0.19 HU in the reconstruction with 140 keV.


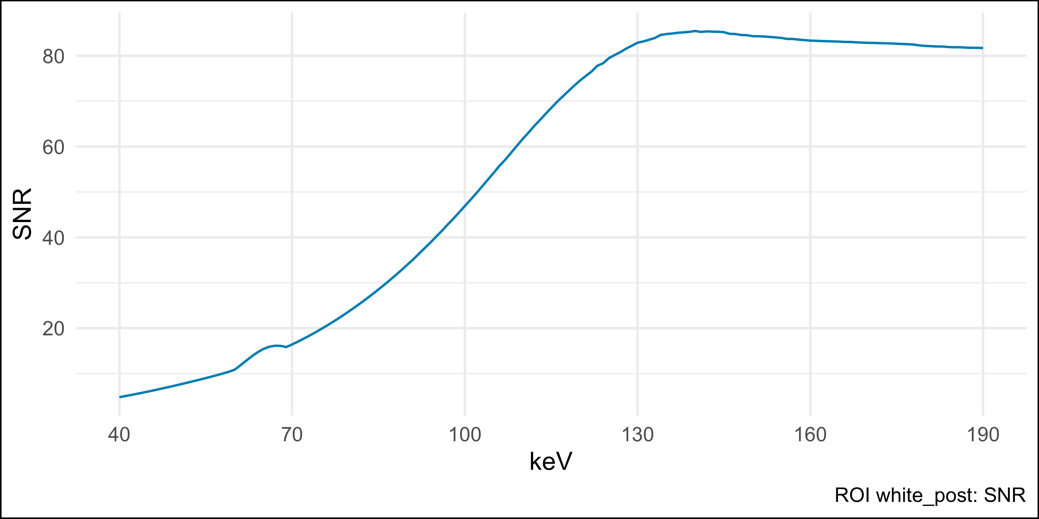


There are significant differences between the SNR at different keV levels (Friedman test corrected p < 0.001). The maximal SNR is 85.46 ± 32.79 in the reconstruction with 140 keV.


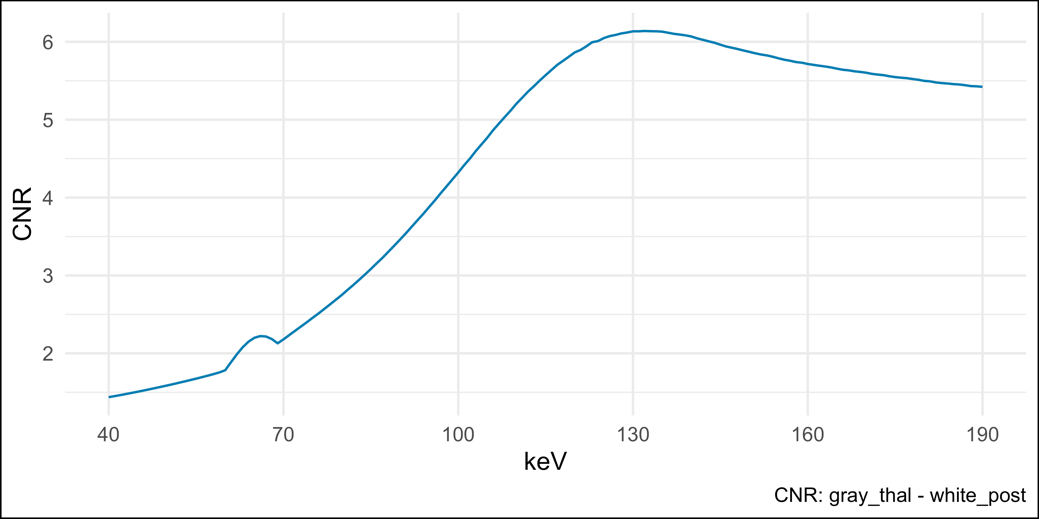


There are significant differences between the CNR at different keV levels (Friedman test corrected p < 0.001). The maximal CNR is 6.14 ± 3.3 in the reconstruction with 132 keV.

The values of signal, noise, SNR and CNR of all keV levels as well as the results of the post hoc testing can be viewed in an a supplemental table (data_image_quality.xlsx).

**ROI (16): Pons between the Petrous Bones**

**
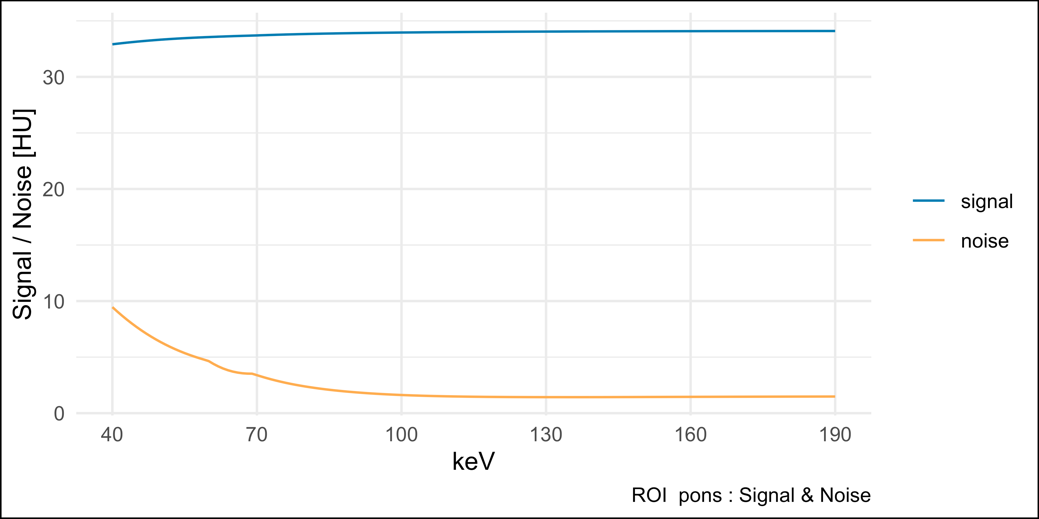
**

There are significant differences between noise (posterior fossa index) at different keV levels (Friedman test corrected p < 0.001). The minimal noise is 1.43 ± 0.59 HU in the reconstruction with 137 keV.

**
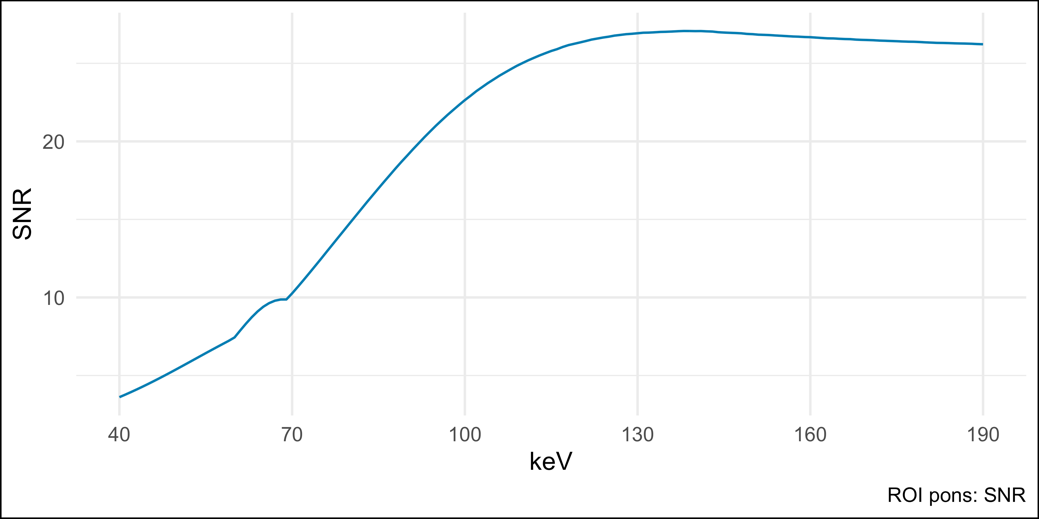
**

There are significant differences between the SNR at different keV levels (Friedman test corrected p < 0.001). The maximal SNR is 27.07 ± 9.44 in the reconstruction with 138 keV.

The values of signal, noise and SNR of all keV levels as well as the results of the post hoc testing can be viewed in an a supplemental table (data_image_quality.xlsx).
